# Template-free synthesis of porous carbon from triazine based polymers and their use in iodine adsorption and CO_2_ capture

**DOI:** 10.1038/s41598-018-20003-1

**Published:** 2018-01-30

**Authors:** Chan Yao, Guoyan Li, Jiku Wang, Yanhong Xu, Limin Chang

**Affiliations:** 1 Key Laboratory of Preparation and Applications of Environmental Friendly Materials of the Ministry of Education, Jilin Normal University, Changchun, 130103 China; 2 Key Laboratory of Functional Materials Physics and Chemistry of the Ministry of Education, Jilin Normal University, Siping, 136000 China

## Abstract

A series of novel triazine-containing pore-tunable carbon materials (NT-POP@800-1-6), which was synthesized via pyrolysis of porous organic polymers (POPs) without any templates. NT-POP@800-1-6 possess moderate BET surface areas of 475–736 m^2^ g^−1^, have permanent porosity and plenty of nitrogen units in the skeletons as effective sorption sites, and display relatively rapid guest uptake of 56–192 wt% in iodine vapour in the first 4 h. In addition, all the samples exhibit the outstanding CO_2_ adsorption capacity of 2.83–3.96 mmol g^−1^ at 273 K and 1.05 bar. Furthermore, NT-POP@800-1-6 show good selectivity ratios of 21.2–36.9 and 3.3–7.5 for CO_2_/N_2_ or CH_4_/N_2_, respectively. We believe that our new building block design provides a general strategy for the construction of triazine-containing carbon materials from various extended building blocks, thereby greatly expanding the range of applicable molecules.

## Introduction

Recently, covalent triazine frameworks (CTFs) as a new subclass of porous organic polymers (POPs) have raised a lot of attention because of their high surface areas, and high thermal and chemical stabilities^1–3^, and represented outstanding performances in a wide range of applications such as gas storage and separation^4,5^, catalysts supports for metals^6,7^, solid base catalyst^8,9^, and sorbent materials^10,11^. Classically, CTFs materials were formed by the trimerization of the cyano groups of aromatic nitriles in salt melts at high temperature, typically in ZnCl_2_ salt melts. Additionally, CTFs were also prepared in trifluoromethanesulfonic acid and microwave heating. However, neither the ionothermal method nor the microwave approach has their disadvantages (such as harsh reaction condition, a limited number of monomers, and difficult to remove catalyst), which limits their practical applications.

The development of potent gas storage systems has been fueled by the demand for highly selective gas capture materials, which is apt to selectively filter out or enrich relevant gases such as carbon dioxide or methane. It is well known that anthropogenic emissions of carbon dioxide are the main source of global warming, which mainly refer to the increase concentration of carbon dioxide have a significant impact on the increased temperature of the earth’s surface. Therefore, people are eager to find a solution to reduce the concentration of carbon dioxide in the atmosphere and to limit its emissions, and to study the ability of capture and storage of new materials. Porous carbon materials as a class of effective storage materials have been received scientific and technological interests because of their large specific surface areas and permanent porosities^12,13^. In general, porous carbon materials were prepared by rigid- or soft-templating methods. Templating essentially involves the replication of one structure into another structure under structural inversion. Templating is a general technique for the formation of nanostructures or porous materials, such as size and shape of the resulting pore structures, which can be easily adjusted by choosing the appropriate template structures. However, it remains a challenge for the synthesis of tunable porous carbon materials without the addition of any templates.

**Figure 1:**
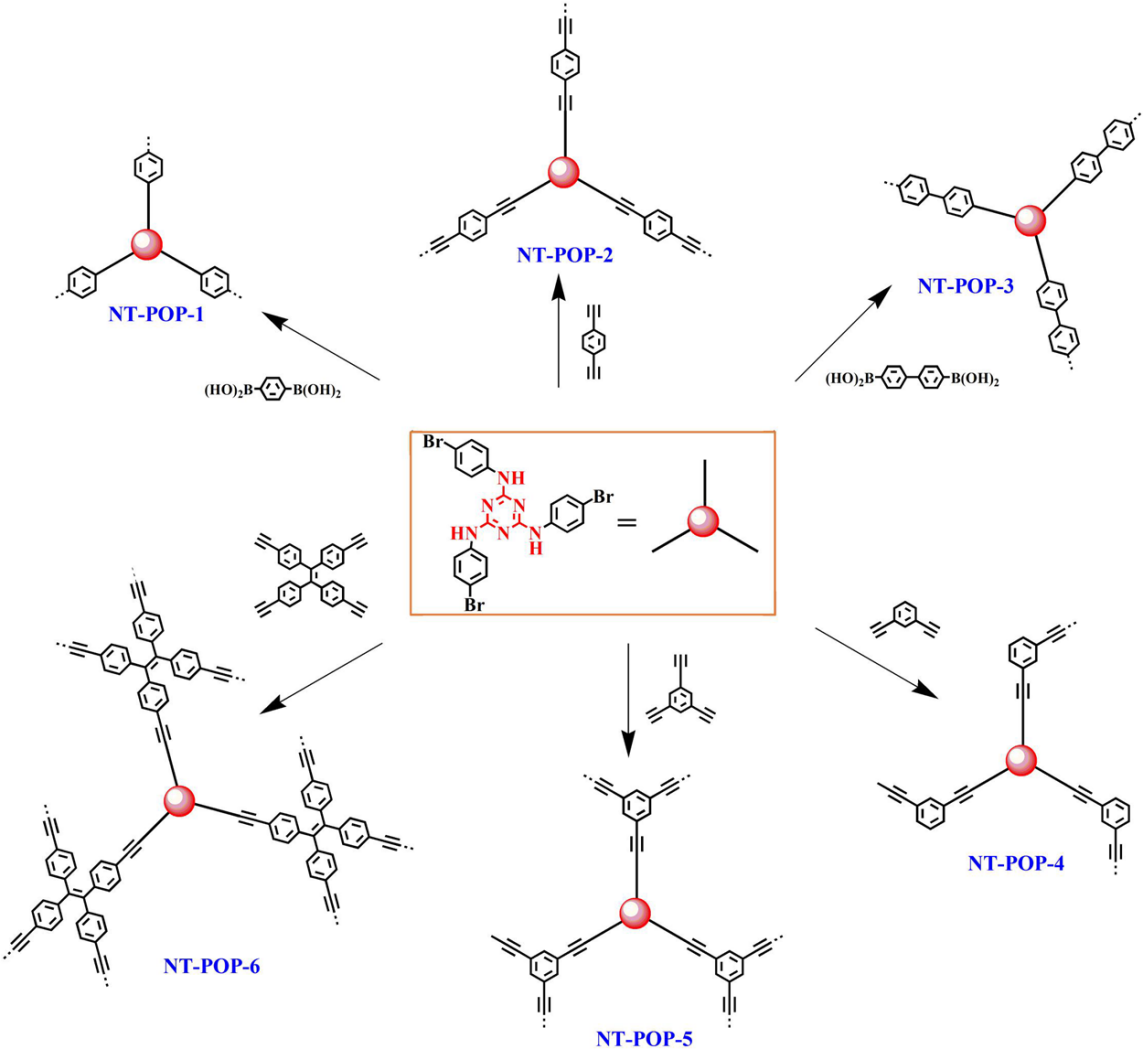
Synthetic route of NT-POP-1-6 microporous adsorbents.

Herein we have developed a building block design concept that allows us to the synthesis of tunable porous carbon materials containing triazine ring without the addition of any templates. Firstly, a series of nitrogen-enriched porous organic polymers (NT-POPs) was synthesized via utilizing rigidity- and flexible- *N*^2^,*N*^4^,*N*^6^-tris(4-bromophenyl)-1,3,5-triazine-2,4,6-triamine (TPTT) as a core building block by a bottom-up strategy (Fig. 1). Subsequent thermal treatment of these NT-POPs precursors at 800 °C, yielded nitrogen-doped porous carbon materials, and these were denoted NT-POP@800-1, -2, -3, -4, -5, and -6, respectively. TPTT is an interesting structural unit for the construction of a range of organic materials because of its rigidity core (triazine ring) and three flexible arms generated from the mutual steric interactions of the peripheral phenyl rings, and it contains a large number of nitrogen groups in the structure. The as-prepared NT-POPs have low specific surface areas (8–90 m^2^ g^−1^) (SI, Figure S1), which may be arised from the interpenetration structures of NT-POPs due to core monomer (TPTT) with flexible arms. The pyrolytic polymers not only have the advantages of high thermal stability compared to usual organic materials, but also possess some properties of CTF materials, and show a greater flexibilities of modifications compared to traditional carbon materials. NT-POP@800-1-6 show the higher surface areas (475–736 m^2^ g^−1^) compared to precursors NT-POPs, retained micropore size, and nitrogen contents of 2.1–4.6%. Previous reports demonstrated that POPs can be used as effective absorbents for safe and long-term capture and storage of iodine or carbon dioxide, not only because of their high surface areas, but also high affinity binding sites (such as ionic bond, phenyl ring, triple bond, enriched π electron, nitrogen-riched or sulfur-riched heteroatom groups) for guest molecules^14–20^. As expected, NT-POP@800-1-6 with the combination of high density nitrogen functional groups and well-defined micropore size exhibit relatively fast iodine capture both in vapor and solution, and excellent CO_2_ uptake and CO_2_/N_2_ selectivity.

## Results

### Synthesis and characterization

All of the polymer networks were synthesized by palladium(0)-catalyzed cross-coupling polycondensation of *N*^2^,*N*^4^,*N*^6^-tris(4-bromophenyl)-1,3,5-triazine-2,4,6-triamine (TPTT) and a number of benzeneboronic monomers or ethynyl monomers. All the reactions were carried out at a fixed reaction temperature and reaction time (120 °C/48 h). Then, the pyrolysis reactions of these precursors (NT-POPs) were carried out on quartz tubes at 800 °C for 2 h under a nitrogen atmosphere, yielded nitrogen-doped porous carbon materials (NT-POP@800-1-6).

The reaction process can be monitored by FT-IR measurements (SI, Figure S2). The disappearance of the strong bands in NT-POP-1-6 skeletons at 1075 cm^−1^ and 522 cm^−1^ were attributed to the breaking of the C–Br bonds, while the second peak close to 2900 cm^−1^, corresponding to –C–H stretching of benzene ring. In addition, a relatively weak peak at approximate 2202 cm^−1^, which referred to –C≡C– stretching of alkynyl moiety of NT-POP-2, 4, 5, and 6, respectively, thus demonstrating the success and completion of the cross-coupling reaction. The elemental analysis results confirmed the C, H, and N contents are close to the theoretical values of the infinite 2D polymers. Further analysis of the polymers by TGA showed that the networks were thermally stable to around 380 °C (SI, Figure S3). After the pyrolysis at 800 °C in a nitrogen flow, the NT-POP-1-6 polymers could be transformed into nitrogen-doped carbon materials (NT-POP@800-1-6). The NT-POP@800-1-6 were obtained in excellent yields up to about 55 wt%.

**Figure 2:**
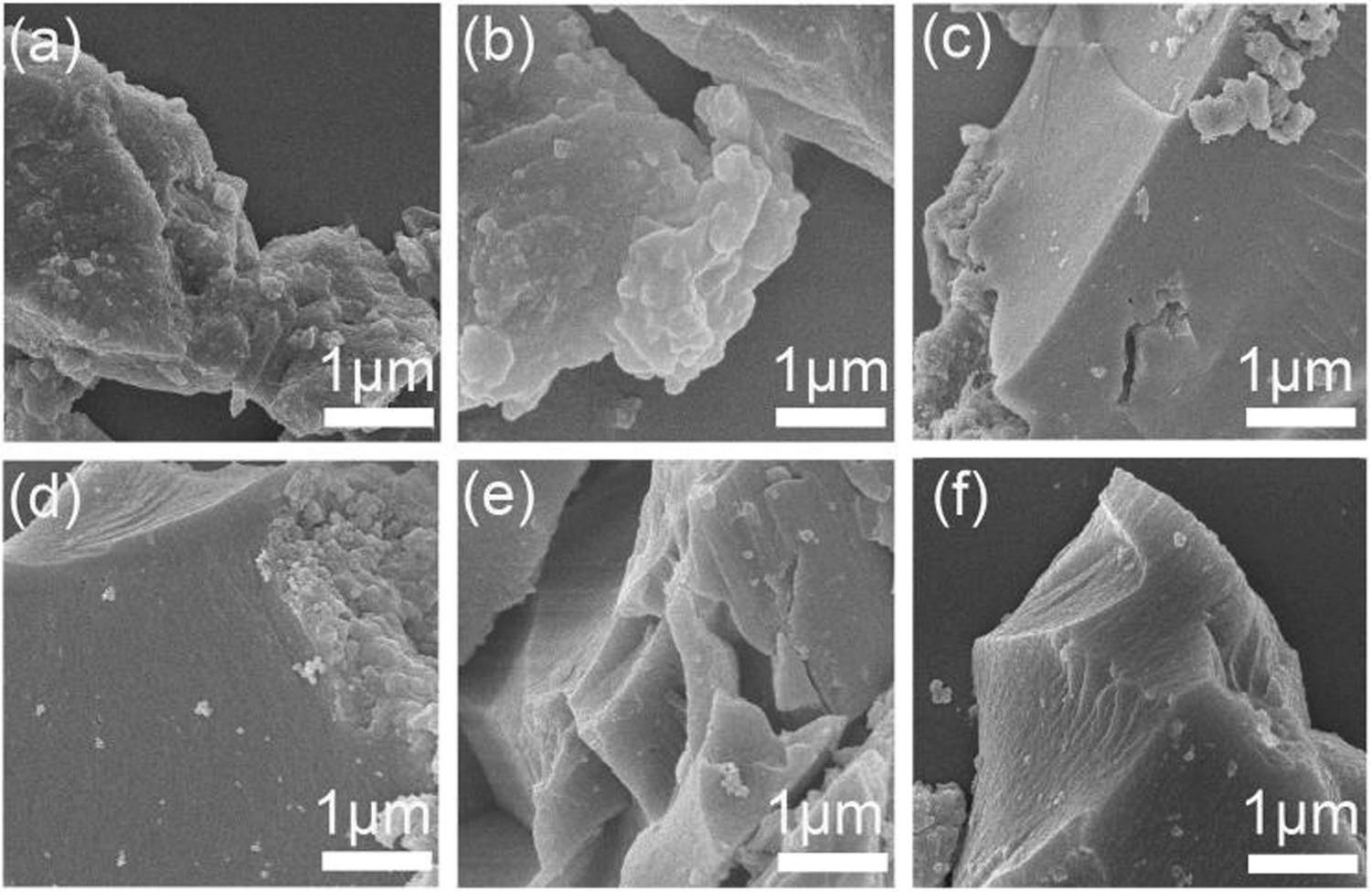
FE-SEM images of (**a**) NT-POP@800-1, (**b**) NT-POP@800-2, (**c**) NT-POP@800-3, (**d**) NT-POP@800-4, (**e**) NT-POP@800-5, and (**f**) NT-POP@800-6, respectively.

The NT-POP@800-1-6 carbon materials are likely to possess a highly porous texture by this highly efficient high temperature pyrolysis method. However, it is difficult to obtain a regular framework via the kinetics controlled irreversible chemistry. Simultaneously, powder X-ray diffraction (PXRD) and transmission electron microscopy (TEM) were carried out to investigate the crystallinity of the samples. The wide-angle PXRD profiles of both NT-POP-1-6 and NT-POP@800-1-6 exhibit the similar shapes without any sharp signals, indicating that they are amorphous random frameworks (SI, Figures S4 and S5). TEM images disclose the absence of long-range order in the NT-POP@800-1-6 samples, which are matched well with those from PXRD analysis (SI, Figure S6). Moreover, the NT-POP@800-1-6 exhibit different morphologies from those of the as-prepared NT-POP-1-6 polymers. SEM analysis reveals NT-POP-1-6 (SI, Figure S7) show a general morphology of the aggregates of particles with different sizes, while NT-POP@800-1-6 display blocky appearances with the sizes more than 1 micron (Fig. 2).

**Figure 3:**
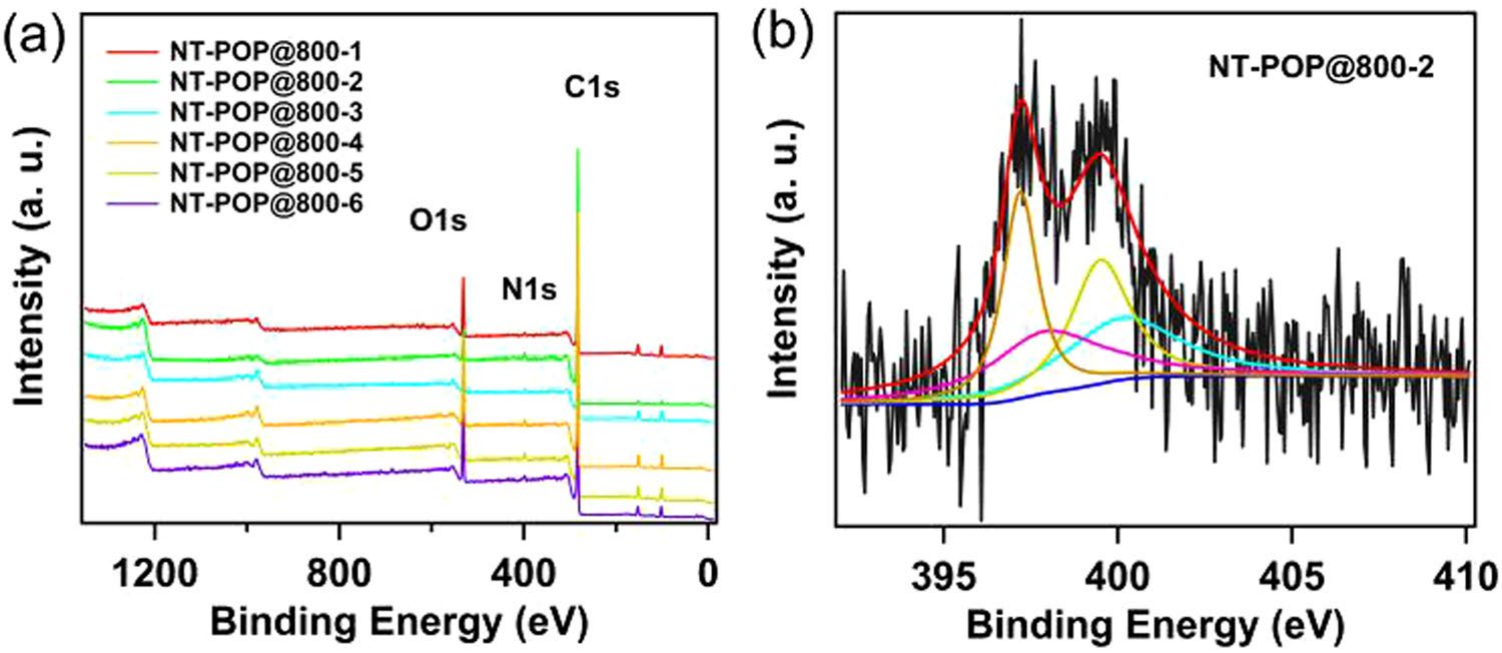
(**a**) XPS survey spectra, (**b**) deconvoluted N1s spectra of NT-POP@800-2.

X-ray photoelectron spectroscopy (XPS) were performed to examine the chemical compositions of the NT-POP@800-1-6. Both survey scan and narrow scan (N1s) were performed. C1s and N1s peaks were observed in the XPS spectra of all the samples (Fig. 3a). The presence of oxygen can be ascribed to carbon materials adsorbed on samples such as O_2_, H_2_O and so on. All of N1s XPS spectra of NT-POP@800-1-6 can be fitted to four main different signals at 396.7, 398.6, 399.2, and 400.1 eV, which are ascribed to 1,3,5-triazine N, pyridinic N, amide or imine, and pyrrolic N, respectively (Figs 3b and S8)^21–23^.

**Figure 4:**
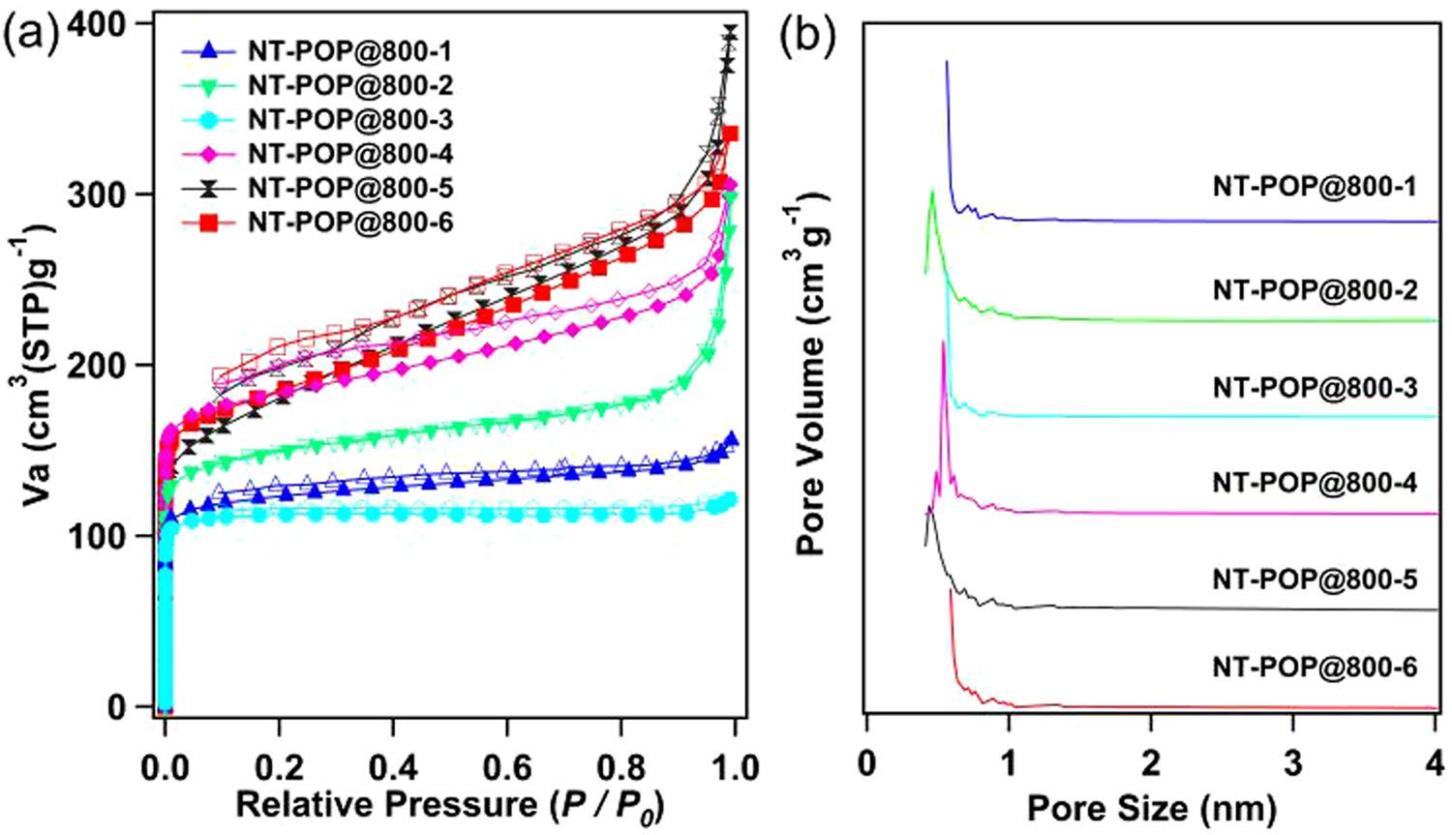
(**a**) Nitrogen sorption curves (filled circles: adsorption, open circles: desorption, STP = standard temperature pressure) and (**b**) pore size distribution.

The porosity of NT-POP-1-6 and NT-POP@800-1-6 were tested by sorption analysis using nitrogen as the sorbate molecule and adsorption/desorption isotherms were collected at 77 K. All of the porous materials exhibited reversible nitrogen sorption isotherm curves (Figs 4a and S1). NT-POP@800-1-6 gave rise to type I isotherms with type IV characters at higher relative pressures (Fig. 4a)^24^, implying that the materials consist of micro- and mesopores. The apparent BET surface areas for the carbon materials (SI, Table S1) were calculated over the relative pressure range *P*/*P*_0_ = 0.015–0.1, which were found to give a positive value of C in the Brunauer-Emmett-Teller (BET) equation. Among the obtained materials, NT-POP@800-4 displayed the highest BET specific surface area of 736 m^2^ g^−1^, followed by NT-POP@800-6 (712 m^2^ g^−1^), NT-POP@800-5 (643 m^2^ g^−1^), NT-POP@800-2 (630 m^2^ g^−1^), NT-POP@800-1 (499 m^2^ g^−1^), and NT-POP@800-3 (475 m^2^ g^−1^). Compared to NT-POP@800-1, -2 and -3, nitrogen gas sorption isotherms of NT-POP@800-4, -5 and -6 showed the obvious H3 hysteresis loops. The gradual increase in nitrogen uptake and the minor hysteresis may be due to the flexible nature of organic materials. Besides that, NT-POP-1 had a low BET surface area of 80 m^2^ g^−1^, followed by 35, 90, 5, 8, and 58 m^2^ g^−1^ for NT-POP-2, 3, 4, 5, and 6, respectively (SI, Figure S1 and Table S1). NT-POP@800-1-6 can enhance the BET surface areas by 5-147 fold than those of the corresponding precursors NT-POP-1-6, respectively (SI, Table S1). These results suggested that the pyrolysis approach allow an improved pore of the non- and low-pore materials.

The pore volumes of the samples were also calculated from the nitrogen isotherms, which were estimated from the amount of gas adsorbed at *P*/*P*_0_ = 0.99. Our results indicated that the total pore volumes of the NT-POP@800-1-6 samples are greatly influenced by the angles of linked-monomers. The total pore volume are 0.239, 0.433, 0.187, 0.463, 0.602, and 0.517 cm^3^ g^−1^ for NT-POP@800-1, 2, 3, 4, 5, and 6, respectively. Furthermore, the pore volumes for the samples are in the order of NT-POP@800-5 > NT-POP@800-6 > NT-POP@800-4 > NT-POP@800-2 > NT-POP@800-1 > NT-POP@800-3. The micropore volumes of the samples calculated by the *t*-method pore volume were 0.044–0.433 cm^3^ g^−1^ for the six nitrogen-doped carbons (SI, Table S1). Previous reports demonstrated that the choice of both linked-monomer length and structure should lead to different pore structures^25,26^. In this work, we do a major research about the monomer structures under the similar reaction conditions. From these results, we found that the geometries of *meta*- and *ortho*-linked monomers can promote higher porosity of polymers than those of *para*-linkaged networks, such as *meta*-linked NT-POP@800-5 possesses the largest pore volume of 0.602 cm^3^ g^−1^, which is larger than those of *para*-linkaged NT-POP@800-1, NT-POP@800-2, and NT-POP@800-3 (SI, Table S1). While for NT-POP@800-3, which is produced from the longest *para*-linkaged 4,4′-biphenyldiboronic acid monomer, shows a minimum pore volume of 0.187 cm^3^ g^−1^. The pore size distributions (PSD) of NT-POPs@800-1-6 samples with the pore widths centering around 0–1.0 nm were calculated by the Saito-Flory method (Fig. 4b). These results revealed that the pyrolysis of POPs without any templates was an efficient route for tuning the pore of carbon materials.

## Discussion

### Gas uptake capacity and separation

**Figure 5:**
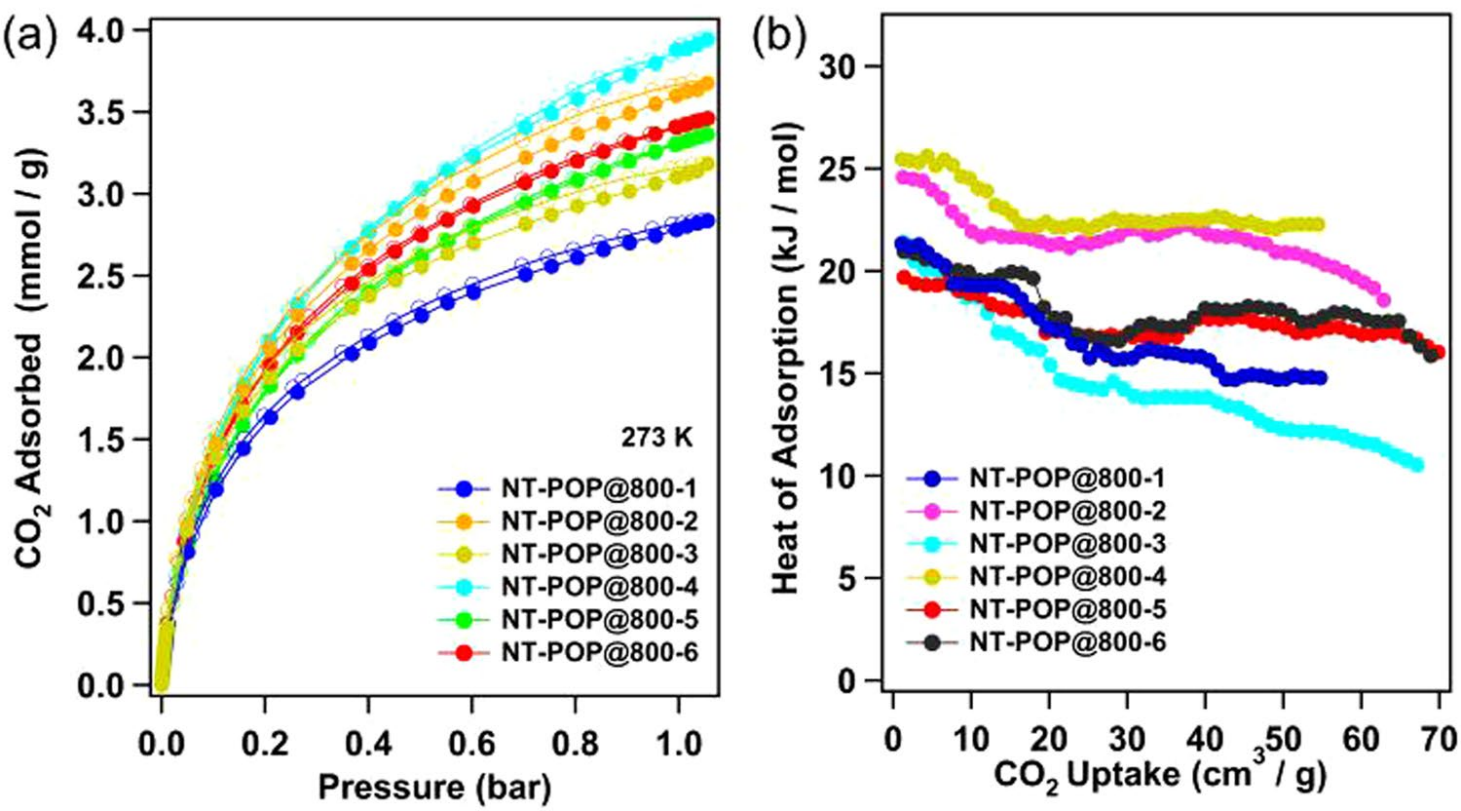
CO_2_ adsorption isotherms collected (**a**) at 273 K and 1.05 bar; (**b**) the isosteric heat of adsorption for NT-POP@800-1-6.

In this case, microstructured polymeric precursors, which contained plenty of nitrogen atoms, and were directly subjected to N_2_ treatment at high temperature, therefore, resulted porous carbon materials possessed much higher nitrogen contents (NT-POPs@800-1-6). To investigate the storage capacity of CO_2_ of the NT-POPs@800-1-6, the isotherms of the samples were measured up to 1.05 bar at 298 K and 273 K, respectively. Of all the carbon materials studied, NT-POP@800-4 exhibited the highest CO_2_ uptake of 3.96 mmol g^−1^ at 273 K and 3.25 mmol g^−1^ at 298 K (Figs 5a and S9), which is compare with some N-doped carbon materials (4.3 mmol g^−1^, *S*_BET_ = 1360 m^2^ g^−1^)^12^. As shown in Fig. 5a, at 273 K and 1.05 bar, the CO_2_ uptakes of porous carbon materials are 2.83, 3.68, 3.19, 3.37, and 3.46 mmol g^−1^ for NT-POP@800-1, NT-POP@800-2, NT-POP@800-3, NT-POP@800-5, and NT-POP@800-6, respectively. Although all the porous carbon materials show moderate surfaces and no saturation state with the measured range of pressures and temperatures, they display significant uptakes of CO_2_, which indicated that the CO_2_ storage of NT-POP@800-1-6 can be enhanced with the pressure increasing or temperature decreasing. In order to compare CO_2_ capture capability with precursor polymers, we performed the CO_2_ uptakes of NT-POP-1-6. In contrast to NT-POP@800-1-6, NT-POP-1, 2, 3, 4, 5, and 6 exhibited the CO_2_ uptakes are 0.51, 0.73, 0.44, 0.49, 0.78, and 0.50 mmol g^−1^ at 273 K and 1.05 bar, respectively (SI, Figure S10 and Table S1). Interestingly, except NT-POP@800-5, other triazine-containing porous carbon materials display much higher CO_2_ capture capability, which are higher 5.0-times than those of the corresponding NT-POPs precursors, respectively, indicating that the NT-POP@800-1-6 can efficiently capture carbon dioxide due to the rich nitrogen groups of carbon materials surface under the same conditions.

More interestingly, NT-POP@800-2 (*S*_BET_ = 630 m^2^ g^−1^) has a lower BET surface area, however, NT-POP@800-2 has a slight less CO_2_ capture capacity of 3.68 mmol g^−1^ at 273 K compare to that of NT-POP@800-4 (3.96 mmol g^−1^, *S*_BET_ = 736 m^2^ g^−1^), which could be attributed to the narrower micropore size in NT-POP@800-2 and high micropore surface area (SI, Table S1). These CO_2_ uptake values are not only higher than those of many microporous polymers with the similar specific surface area but also comparable to the reported large surface area of porous aromatic frameworks under the same conditions, such as CMP-1-COOH (1.60 mmol g^−1^, *S*_BET_ = 522 m^2^ g^−1^)^27^, PECONF-1 (1.86 mmol g^−1^, *S*_BET_ = 499 m^2^ g^−1^)^28^, HPF-1 (2.80 mmol g^−1^, *S*_BET_ = 576 m^2^ g^−1^)^29^, COF-1 (2.32 mmol g^−1^, *S*_BET_ = 750 m^2^ g^−1^)^30^, CMP-1-(OH)_2_ (1.80 mmol g^−1^, *S*_BET_ = 1043 m^2^ g^−1^)^27^, PAF-4 (2.41 mmol g^−1^, *S*_BET_ = 2246 m^2^ g^−1^)^31^, COF-102 (1.56 mmol g^−1^, *S*_BET_ = 3620 m^2^ g^−1^)^30^, PAF-1 (2.05 mmol g^−1^, *S*_BET_ = 5460 m^2^ g^−1^)^32^, *fl*-CTF-400 (4.13 mmol g^−1^, *S*_BET_ = 2862 m^2^ g^−1^)^33^, and azolyl-carboxylate MOFs (2.96-3.02 mmol g^−1^, *S*_BET_ = 548-722 m^2^ g^−1^)^34^. The analysis results clearly demonstrated that the combination of high density nitrogen functional groups and well-defined micropore size in the porous carbon materials can lead to excellent CO_2_ adsorption capacities. We calculated the CO_2_ isosteric enthalpy of adsorption (*Q*_st_) in order to provide a better understanding of the CO_2_ uptake properties, which was from Clausius-Clapeyron equation using adsorption data collected at 273 K and 298 K (Fig. 5b). From the curves, we found that all of the samples showed the isosteric heats of CO_2_ adsorption from 25.4 to 19.4 kJ mol^−1^ at the near zero coverage. Then, the heat values of NT-POP@800-1-6 dropped to about 22.3-11.2 kJ mol^−1^ with the loading increasing.

To examine the separation ability of NT-POP@800-1-6 for different gases, CH_4_ and N_2_ sorption experiments were carried out at 273 K and 1.05 bar, respectively (SI, Figure S11). The CO_2_/N_2_ and CH_4_/N_2_ selectivities of NT-POP@800-1-6 were calculated by using the ideal adsorbed solution theory (IAST). At 273 K and 1.05 bar, the CO_2_/N_2_ and CH_4_/N_2_ adsorption selectivities for the NT-POP@800-1-6 carbon samples are calculated to be 21.2–36.9 and 3.3–7.5 via the IAST method, respectively (SI, Table S1 and Figure S12).

### Iodine capture

**Figure 6:**
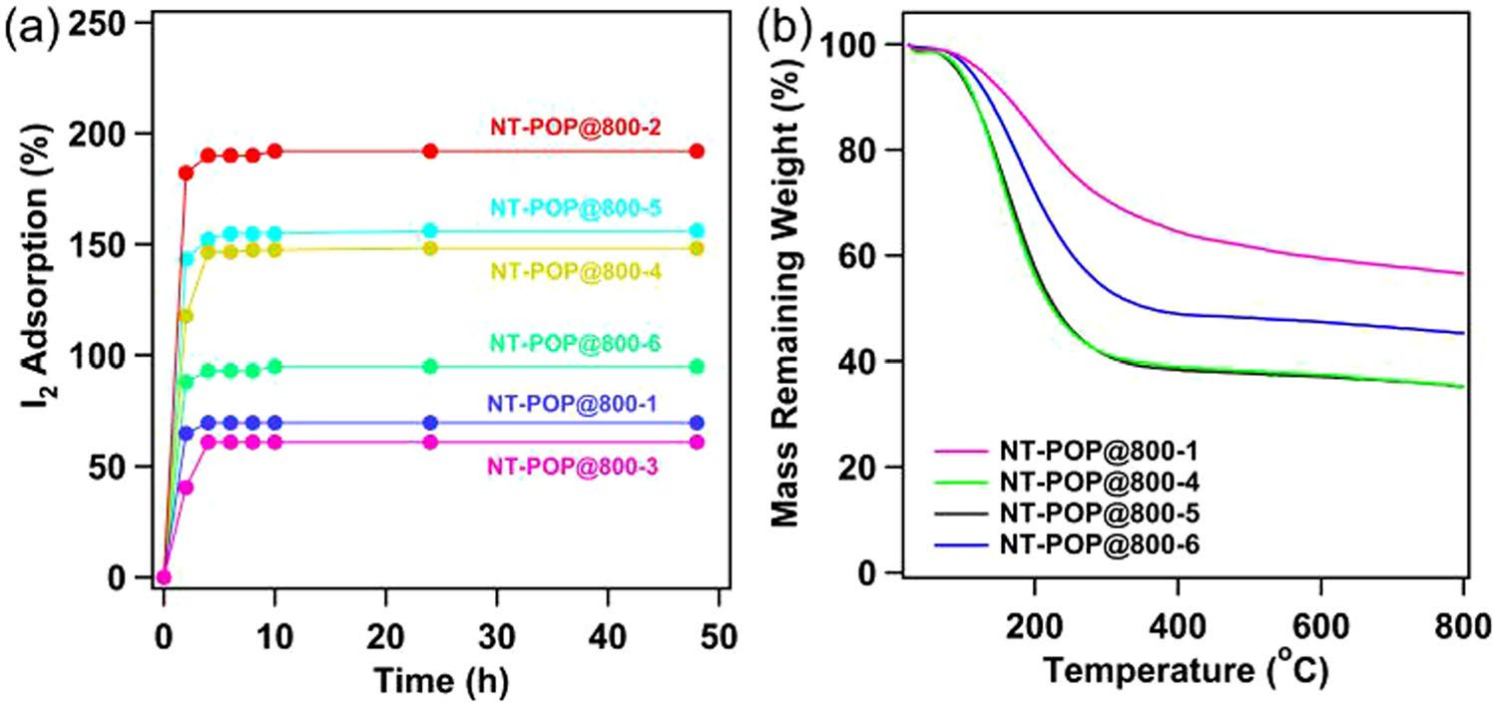
(**a**) Gravimetric uptake of iodine as a function of time at 350 K; (**b**) TGA trace of I_2_@NT-POP@800.

The performances for capture of iodine vapor of this series of NT-POP@800-1-6 were evaluated by directly gravimetric measurements. The samples of NT-POP@800-1-6 were pre-weighted and kept in small weighing bottles respectively, which were located in a sealed container in the presence of solid iodine. The iodine sublimed into the porous absorbent over time at 350 K and ambient pressure, which is close to the actual nuclear-fuel reprocessing conditions. Due to the color of porous carbon samples is deep black, therefore, we don’t find the color of the samples change with the time progressed. Figure 6a showed that gravimetric measurements were taken at various time intervals during the iodine uptake, and the results indicated that the mass of iodine uptake significantly increased in the initial 4 h and reached a platform thereafter, suggesting a saturated adsorption was reached. The saturated I_2_ loading of NT-POP@800-1, 2, 3, 4, 5, and 6 are 68, 192, 56, 149, 152, and 95 wt.%, respectively. Among them, NT-POP@800-2 shows the highest iodine adsorption value in the obtained carbons, NT-POP@800-4 and NT-POP@800-5 have a equal results, while NT-POP@800-1 displays the lowest iodine adsorption value. NT-POP@800-2, -4 and -5, although they have lower specific surface areas and pore volumes, however, the iodine uptakes of the carbon materials are comparable to those of PAF-1 (*S*_BET_ = 5600 m^2^ g^−1^, 186 wt.%)^32,35^, NiP-CMP (*S*_BET_ = 2600 m^2^ g^−1^, 202 wt.%)^36^, CMPN-3 (*S*_BET_ = 1368 m^2^ g^−1^, 208 wt.%)^37^, JUC-Z2 (*S*_BET_ = 2081 m^2^ g^−1^, 144 wt.%)^38,39^, and other porous networks^40–47^. This result suggested that the iodine adsorption capacity of porous materials is not only dependent on specific surface area, but also high affinity functional groups^14–17^. The thermogravimetric analysis (TGA) of the I_2_-loaded NT-POP@800-1-6 samples revealed a significant weight loss from 90 to 300 °C (Fig. 6b), the calculated iodine mass loss were 75, 152, 158, and 101 wt.% for NT-POP@800-1, NT-POP@800-4, NT-POP@800-5, and NT-POP@800-6, respectively, which are close to the saturated adsorption value.

Moreover, the absorbed iodine can be easily removed by immersing the I_2_-loaded samples in ethanol at room temperature. The recycling is an important parameter for their practical applications. We tested three samples and found the samples could be efficiently recycled and reused for five cycles without significant loss of iodine uptakes (SI, Figure S13). X-ray photoelectron spectroscopy (XPS) of all the I_2_-loaded NT-POP@800-1-6 implyed that the coexistence of elemental iodine and triiodide ion, suggesting a hybrid of physisorption and chemisorption (SI, Figure S14). Interestingly, for any NT-POP@800-1-6 materials, the signal strength attributed to I_3_- is almost the same as that of neural I_2_, indicating physisorption and chemisorption are almost equally important in the I_2_ capture process by NT-POP@800 materials. Unlike NT-POP@800-1, -3, -4, -5 and -6, NT-POP@800-2 exhibits the stronger signal of I_3_- compared to neural I_2_.

The capability of trapping iodine of NT-POP@800-1-6 was also tested in organic solution at ambient conditions. The uptake was monitored by UV/Vis spectroscopy. The iodine solution (4 mg mL^−1^, 3 mL) containing 30 mg of NT-POP@800 samples was kept for different periods up to 48 h. The purple color of the iodine solution gradually changed from deep purple to light purple and finally to paler (SI, Figure S15). The UV/Vis absorption intensity of the samples was decreased with the prolonged action time (SI, Figure S16). The adsorption kinetics of iodine at 25 °C were presented, as illustrated in Figure S17. The iodine sorption can be divided into two stages: the adsorption capacity for iodine quickly increased during the first 2 h, and a slowly increased iodine uptake until equilibrium. The adsorption performances of NT-POP@800-1-6 were analyzed using Lagergren pseudo-first-order and pseudo-first-order kinetic models, respectively (SI, Table S2, Figures S18 and S19). The adsorption kinetics fit the pseudo-first-order kinetic model, good linear fits with correlation coefficient *R*^2^ values of 0.9786, 0.9593, 0.9601, 0.9517, 0.9877, and 0.9432 for NT-POP@800-1, NT-POP@800-2, NT-POP@800-3, NT-POP@800-4, NT-POP@800-5, and NT-POP@800-6, respectively. Finally, all the carbon materials exhibited the removal efficiencies of up to 99% in the iodine solutions with a concentration of 4 mg mL^−1^. Also, the adsorption isotherm is a significant factor in determining the saturated adsorption capacity (SI, Table S3, Figures S20 and S21). The adsorption plot of equilibrium concentration versus adsorption capacity showed that the two adsorption stages. Firstly, the equilibrium absorption linearly increased with the increase of iodine concentration. Compared with the Freundlich equation, the fitting of Langmuir equation is more in line with the experimental curve, the calculation results suggested that a monolayer adsorption behavior for iodine on the surface of NT-POP@800-1-6 samples. The adsorption reached the maximum uptake without relation to the increasing iodine concentration. From the kinetic studies, NT-POP@800-2, -3, and -4 represent a high iodine uptake of 1191, 970, and 1202 mg g^−1^, respectively.

## Conclusion

In summary, we have developed a building block for designing covalent triazine framework and achieved a series of novel nitrogen-enriched pore-tunable carbon materials via pyrolysis of POPs without any templates. The structures of NT-POP@800-1-6 carbon materials were well characterized and discussed. NT-POP@800-1-6 display relatively high-speed iodine capture both in vapor and solution, and excellent CO_2_ uptake and CO_2_/N_2_ selectivity. Furthermore, at 273 K and 1.05 bar, NT-POP@800-4 exhibits the highest CO_2_ uptake of 3.96 mmol g^−1^, the rest polymers are in the range of 2.83-3.68 mmol g^−1^, and the samples display good selectivities of 21.2–36.9 and 3.3–7.5 for CO_2_/N_2_ or CH_4_/N_2_, respectively. The BET surface areas and CO_2_ uptakes of NT-POP@800-1-6 are 5-147 times and 3.8-6.6 times as value as corresponding precursors, respectively. The results indicated that the present strategy provides a facile route for synthesis of triazine-containing networks and modifying the pore structures of carbon materials. We believed that the building block concept will greatly expand the range of applicable molecules for synthesis of triazine-containing porous carbons with tailor-made pore.

## Methods

### Synthesis of NT-POP-1

*N*^2^,*N*^4^,*N*^6^-tris(4-bromophenyl)-1,3,5-triazine-2,4,6-triamine (147.8 mg, 0.25 mmol) and 1,4-phenylenediboronic acid (62.2 mg, 0.375 mmol) were put into a 50 mL two-necked round-bottom flask, then the flask was exchanged 3 cycles under vacuum/N_2_. Then added 10 mL DMF, the flask was further degassed by freeze-pump-thaw for 3 times. When the solution had reached reaction temperature, a slurry of tetrakis(triphenylphesphine)palladium (0) (17.3 mg, 0.015 mmol) in 6 mL DMF and potassium carbonate (207 mg, 1.5 mmol) in 4 mL distilled water were added, and the reaction was stirred at 120 °C under nitrogen for 48 h. The solid product was collected by filtration and washed well with THF, methanol, acetone, and water for 4 times, respectively. Further purification of the polymer was carried out by Soxhlet extraction with methanol, and THF for 24 h, respectively, to give NT-POP-1 as brown powder (90.8% yield). Elemental Analysis (%) C 80.81, H 4.69, N 14.50; Found C 78.2, H 4.15, N 13.2.

### Synthesis of NT-POP-2

*N*^2^,*N*^4^,*N*^6^-tris(4-bromophenyl)-1,3,5-triazine-2,4,6-triamine (147.8 mg, 0.25 mmol) and 1,4-diethynylbenzene (70.6 mg, 0.56 mmol) were put into a 50 mL two-necked round-bottom flask, then the flask was exchanged 3 cycles under vacuum/N_2_. Then added 2 mL DMF and 2 mL triethylamine (Et_3_N), the flask was further degassed by freeze-pump-thaw for 3 times. When the solution had reached reaction temperature, a slurry of tetrakis(triphenylphesphine)palladium (0) (17.3 mg, 0.015 mmol) in 1 mL DMF and copper (I) iodide (2.7 mg, 0.015 mmol) in 1 mL Et_3_N were added, and the reaction was stirred at 120 °C under nitrogen for 48 h. The solid product was collected by filtration and washed well with THF, methanol, acetone, and water for 4 times, respectively. Further purification of the polymer was carried out by Soxhlet extraction with methanol, and THF for 24 h, respectively, to give NT-POP-2 as yellowish powder (92.5% yield). Elemental Analysis (%) C 84.63, H 3.76, N 11.61; Found C 82.08, H 3.05, N 10.55.

### Synthesis of NT-POP-3

*N*^2^,*N*^4^,*N*^6^-tris(4-bromophenyl)-1,3,5-triazine-2,4,6-triamine (147.8 mg, 0.25 mmol) and 4,4′-biphenyldiboronic acid (90.7 mg, 0.375 mmol) were put into a 50 mL two-necked round-bottom flask, then the flask was exchanged 3 cycles under vacuum/N_2_. Then added 10 mL DMF, the flask was further degassed by freeze-pump-thaw for 3 times. When the solution had reached reaction temperature, a slurry of tetrakis(triphenylphesphine)palladium (0) (17.9 mg, 0.015 mmol) in 6 mL DMF and potassium carbonate (20.7 mg, 0.15 mmol) in 4 mL distilled water were added, and the reaction was stirred at 120 °C under nitrogen for 48 h. The solid product was collected by filtration and washed well with THF, methanol, acetone, and water for 4 times, respectively. Further purification of the polymer was carried out by Soxhlet extraction with methanol, and THF for 24 h, respectively, to give NT-POP-3 as light gray powder (94.3% yield). Elemental Analysis (%) C 84.73, H 4.87, N 10.40; Found C 81.34, H 4.05, N 9.12.

### Synthesis of NT-POP-4

*N*^2^,*N*^4^,*N*^6^-tris(4-bromophenyl)-1,3,5-triazine-2,4,6-triamine (147.8 mg, 0.25 mmol) and 1,3-diethynylbenzene (70.6 mg, 0.56 mmol) were put into a 50 mL two-necked round-bottom flask, then the flask was exchanged 3 cycles under vacuum/N_2_. Then added 2 mL DMF and 2 mL triethylamine (Et_3_N), the flask was further degassed by freeze-pump-thaw for 3 times. When the solution had reached reaction temperature, a slurry of tetrakis(triphenylphesphine)palladium (0) (17.3 mg, 0.015 mmol) in 1 mL DMF and copper (I) iodide (2.7 mg, 0.015 mmol) in 1 mL Et_3_N were added, and the reaction was stirred at 120 °C under nitrogen for 48 h. The solid product was collected by filtration and washed well with THF, methanol, acetone, and water for 4 times, respectively. Further purification of the polymer was carried out by Soxhlet extraction with methanol, and THF for 24 h, respectively, to give NT-POP-4 as yellow powder (88.7% yield). Elemental Analysis (%) C 84.63, H 3.76, N 11.61; Found C 82.34, H 3.22, N 10.89.

### Synthesis of NT-POP-5

*N*^2^,*N*^4^,*N*^6^-tris(4-bromophenyl)-1,3,5-triazine-2,4,6-triamine(147.8 mg, 0.25 mmol) and 1,3,5-triethynylbenzene (56.6 mg, 0.375 mmol) were put into a 50 mL two-necked round-bottom flask, then the flask was exchanged 3 cycles under vacuum/N_2_. Then added 2 mL DMF and 2 mL triethylamine (Et_3_N), the flask was further degassed by three freeze-pump-thaw cycles, purged with N_2_. When the solution had reached reaction temperature, a slurry of tetrakis(triphenylphosphine)palladium (0) (17.3 mg, 0.015 mmol) in 1 mL DMF and copper (I) iodide (2.7 mg, 0.015 mmol) in 1 mL Et_3_N were added, and the reaction was stirred at 120 °C under nitrogen for 48 h. The solid product was collected by filtration and washed well with THF, methanol, acetone, and water for 4 times, respectively. Further purification of the polymer was carried out by Soxhlet extraction with methanol, and THF for 24 h, respectively, to give NT-POP-5 as yellow solid (93.6% yield). Elemental Analysis (%) C 86.35, H 3.05, N 10.60; Found C 83.38, H 3.12, N 9.28.

### Synthesis of NT-POP-6

*N*^2^,*N*^4^,*N*^6^-tris(4-bromophenyl)-1,3,5-triazine-2,4,6-triamine (147.8 mg, 0.25 mmol) and 1,1,2,2-tetrakis(4-ethynylphenyl)ethane (106.5 mg, 0.28 mmol) were put into a 50 mL two-necked round-bottom flask, then the flask was exchanged 3 cycles under vacuum/N_2_. Then added to 2 mL DMF and 2 mL triethylamine (Et_3_N), the flask was further degassed by freeze-pump-thaw for 3 times. When the solution had reached reaction temperature, a slurry of tetrakis(triphenylphesphine)palladium (0) (17.3 mg, 0.015 mmol) in 1 mL DMF and copper (I) iodide (2.7 mg, 0.015 mmol) in1 mL Et_3_N was added, and the reaction was stirred at 120 °C under nitrogen for 48 h. The solid product was collected by filtration and washed well with THF, methanol, acetone, and water for 4 times, respectively. Further purification of the polymer was carried out by Soxhlet extraction with methanol, and THF for 24 h, respectively, to give NT-POP-6 as brownish black powder (91.7% yield). Elemental Analysis (%) C 90.92, H 3.91, N 5.17; Found C 87.08, H 3.12, N 5.44.

### Synthesis of NT-POP@800: Template-free Pyrolysis of NT-POP-1-6

The pyrolysis reactions of the NT-POP-1-6 were carried out on quartz tubes in an electricfurnace under a nitrogen atmosphere. The NT-POP-1, 2, 3, 4, 5, and 6 samples were heated from the room temperature to 800 °C with a heating rate of 3 °C/min, then pyrolyzed at 800 °C for 2 h in nitrogen gas (400 sccm), respectively. The pyrolysis reactions at 800 °C in nitrogen gas were denoted to NT-POP@800-1, NT-POP@800-2, NT-POP@800-3, NT-POP@800-4, NT-POP@800-5, and NT-POP@800-6, respectively.

## Electronic supplementary material

Supplementary Information
