# Supplementary material for: Template-free synthesis of porous carbon from triazine based polymers and their use in iodine adsorption and CO2 capture
Source: Sci Rep. 2018 Jan 30;8:1867. doi: 10.1038/s41598-018-20003-1 (PMC5789819; doi:10.1038/s41598-018-20003-1)
Supplement: Supplementary file 1 — Supplementary Information [file 41598_2018_20003_MOESM1_ESM.doc]

**Supporting Information**

**Template-free synthesis of porous carbon from triazine based polymers and their use in iodine adsorption and CO_2_ capture**

Chan Yao,^a^ Guoyan Li,^a^ Jiku Wang,^a^ Yanhong Xu*^a,b^ and Limin Chang*^a^

Corresponding Author:

Professor Yan-Hong Xu

^a^Key Laboratory of Preparation and Applications of Environmental Friendly Materials (Jilin Normal University), Ministry of Education, Changchun, 130103, China

^b^Key Laboratory of Functional Materials Physics and Chemistry of the Ministry of Education, Jilin Normal University, Siping 136000, China

Email: [xuyh198@163.com](mailto:xuyh@jlnu.edu.cn)

**Contents**

**Section A. Materials and Methods**

**Section B. Synthetic Procedures**

**Section C. Nitrogen Sorption Curves**

**Section D. FT-IR Spectra**

**Section E.TGA Curves**

**Section F. PXRD Curves**

**Section G. HR-TEM Images**

**Section H. FE-SEM Images**

**Section I. XPS Spectra**

**Section J. Gas Adsorption Isotherms**

**Section K. Gas Selectivity Analyses**

**Section L. Iodine Capture Analyses**

**Section M. Supporting References**

**Section A. Materials and Methods**

*N*^2^,*N*^4^,*N*^6^-tris(4-bromophenyl)-1,3,5-triazine-2,4,6-triamine (TPTT) was purchased from Chemsoon Co. Ltd.. 1,3,5-Triethynylbenzene and 1,3-diethynylbenzene were purchased from TCI. Tetrakis(triphenylphosphine)palladium(0), copper(I) iodide, 1,4-diethynylbenzene, 4,4'-biphenyldiboronic acid, and 1,4-phenylenediboronic acid were also purchased from Aladdin. 1,1,2,2-tetrakis(4-ethynylphenyl)ethene was synthesized according to the literature. All the solvents used were purchased from Aladdin.

^1^H NMR spectra were recorded on Bruker AvanceIII models HD 400NMR spectrometers, where chemical shifts (δ in ppm) were determined with a residual proton of the solvent as standard. Fourier transform Infrared (FT-IR) spectra were recorded on a Perkin-elmer spectrum one model FT-IR-frontier infrared spectrometer. Field-emission scanning electron microscopy (FE-SEM) images were performed on a JEOL model JSM-6700 operating at an accelerating voltage of 5.0 kV. The samples were prepared by drop-casting a THF suspension onto mica substrate and then coated with gold. High-resolution transmission electron microscopy (HR-TEM) images were obtained on a JEOL model JEM-3200 microscopy. Powder X-ray diffraction (PXRD) data were recorded on a Rigaku model RINT Ultima III diffractometer by depositing powder on glass substrate, from 2θ = 1.5° up to 60° with 0.02° increment. The elemental analysis was carried out on a EuroEA-3000. TGA analysis was carried out using a Q5000IR analyser (TA Instruments) with an automated vertical overhead thermobalance. Before measurement, the samples were heated at a rate of 5 °C min^-1^ under a nitrogen atmosphere. X-ray photoelectron spectra (XPS) were recorded on an ESCALAB250Xi electron spectrometer (Thermo FisherScientific Inc., Waltham, MA, USA). Nitrogen sorption isotherms were measured at 77 K with ASIQ (iQ-2) volumetric adsorption analyzer. Before measurement, the samples were degassed in vacuum at 150 °C for 12 h. The Brunauer-Emmett-Teller (BET) method was utilized to calculate the specific surface areas and pore volume. BET surface areas were calculated over the relative pressure range 0.015-0.1 *P*/*P*_0_. Pore size distributions were calculated by the Saito-Flory (SF) method from the nitrogen adsorption branch using a cylindrical pore size model. Carbon dioxide, methane and nitrogen sorption isotherms were measured at 298 K or 273 K with a Bel Japan Inc. model BELSORP-max analyzer, respectively.

Carbon dioxide sorption isotherms were measured at 298 K and 273 K with a Bel Japan Inc. model BELSORP-max analyzer, respectively. Before measurement, the samples were also degassed in vacuum at 120 °C for more than 10 h.

Methane and nitrogen sorption isotherms were measured at 273 K with a Bel Japan Inc. model BELSORP-max analyzer, respectively. Before measurement, the samples were also degassed in vacuum at 120 °C for more than 10 h.

**Section B. Synthetic Procedures**

**Synthesis of *p*-Tetrabromotetraphenylethene (TBTPE)** ^S1^

Powdered 1,1,2,2-tetraphenylethene (5.00 g, 15.0 mmol) was treated with bromine (7.50 mL, 0.15 mol) and the mixture was kept for 16 h at room temperature. The resulting solid was dissolved in hot toluene (120 mL), concentrated to about 20 mL, and the precipitate was isolated. Purification using flash chromatography on SiO_2_ (hexanes/CH_2_Cl_2_, 20:1 in vol.) gives TBTPE as a colorless solid (5.94 g) in 61% yield. ^1^H NMR (CDCl_3_, 400 MHz): *δ* (ppm) 7.26 (d, 8H), 6.85 (d, 8H).

**Synthesis of 1,1,2,2-tetrakis[4-(trimethylsilylethynyl)phenyl]ethene**^S2^

TBTPE (1g, 1.54 mmol) and PdCl_2_(PPh_3_)_2_ (25.2 mg, 0.036 mmol) and CuI (3.6 mg, 0.0185 mmol) were put in to a 100 mL round-bottom flask, then the flask exchanged 3 cycles under vacuum/N_2_, then added 50 mL diethylamine. The flask was degassed by freeze-pump-thaw for 3 times, then warmed to R.T., refilled with N_2_, trimethylsilylacetylene(1 mL, 7.392 mmol) was slowly added via a syringe. The mixture was heated at 50 °C for 15 h. After this reaction mixture was cooled to room temperature, concentrated to about 10 mL. The crude product was purified by silica gel column chromatography using hexane as eluent. A white solid was obtained (72% yield). ^1^H NMR (CDCl_3_, 400 MHz): *δ* (ppm): 7.23 (d, 8H), 6.92 (d, 8H), 0.26 (s, 36H).

**Synthesis of 1,1,2,2-tetrakis(4-ethynylphenyl)ethene**^S2^

1,1,2,2-Tetrakis(4-(trimethylsilylethynyl)phenyl)ethene (1.5 g, 2.1 mmol) and THF (40 mL) were placed were put into a 250 mL round-bottom flask. Then, KOH (1.8 g, 32 mmol) dissolved in 40 mL of methanol was added. The mixture was stirred at room temperature overnight. After most of the solvent was evaporated, 100 mL of 1 M aqueous HCl solution was added and the mixture extracted with dichloromethane three times. The organic phases were combined and washed with water and brine and then dried over MgSO_4_. After filtration and solvent evaporation, the crude product was purified by a silica gel column chromatography using hexane/dichloromethane (100:1 by volume) mixture as eluent. A yellow solid was obtained (81% yield). ^1^H NMR (400 MHz, CDCl_3_ ): *δ* (ppm) 7.28 (d, 8H), 6.93 (d, 8H), 3.06 (s, 4H).

**Table S1︱Porosity properties and gas uptake for the polymers.**

| Polymers | *S*_BET_^a^  /m^2^ g^-1^ | *S*_micro_^b^  /m^2^ g^-1^ | V_total_^c^  /cm^3^ g^-1^ | V_micro_  /cm^3^ g^-1^ | CO_2_  At 273 K/mmol g^-1^ | CO_2_  at 298 K/mmol g^-1^ | CO_2_/N_2_^d^  selectivity  at 273 K | CH_4_/N_2_^d^  selectivity  at 273 K |
| --- | --- | --- | --- | --- | --- | --- | --- | --- |
| **NT-POP@800-1** | 499 | 367 | 0.239 | 0.107 | 2.83 | 2.5 | 21.2 | 3.3 |
| **NT-POP@800-2** | 630 | 537 | 0.433 | 0.321 | 3.68 | 3.06 | 25.9 | 4.2 |
| **NT-POP@800-3** | 475 | 403 | 0.187 | 0.043 | 3.19 | 2.38 | 29.8 | 3.7 |
| **NT-POP@800-4** | 736 | 474 | 0.463 | 0.294 | 3.96 | 3.25 | 36.9 | 7.5 |
| **NT-POP@800-5** | 643 | 394 | 0.602 | 0.433 | 3.37 | 2.97 | 26.1 | 3.9 |
| **NT-POP@800-6** | 712 | 510 | 0.517 | 0.321 | 3.46 | 2.78 | 28.7 | 3.7 |
| **NT-POP-1** | 80 | -^e^ | - | - | 0.51 | - | - | - |
| **NT-POP-2** | 35 | - | - | - | 0.73 | - | - | - |
| **NT-POP-3** | 90 | - | - | - | 0.44 | - | - | - |
| **NT-POP-4** | 5 | - | - | - | 0.49 | - | - | - |
| **NT-POP-5** | 8 | - | - | - | 0.78 | - | - | - |
| **NT-POP-6** | 58 | - | - | - | 0.50 | - | - | - |

*^a^*Brunauer-Emmett-Teller surface area. *^b^*Total pore volume determined from the N_2_ isotherm at *P*/*P*_0_=0.995. *^c^*Micro-pore volume determined from the N_2_ isotherm at P/P_0_ = 0.050. *^d^*Selectivity was calculated by IAST method for CO_2_/N_2_ (at mole ratio of 15/85 ) and CH_4_/N_2_ (at mole ratio of 50/50) at 273 K. ^e^ Not mentioned.

**Section C. Nitrogen Sorption Curves**

**
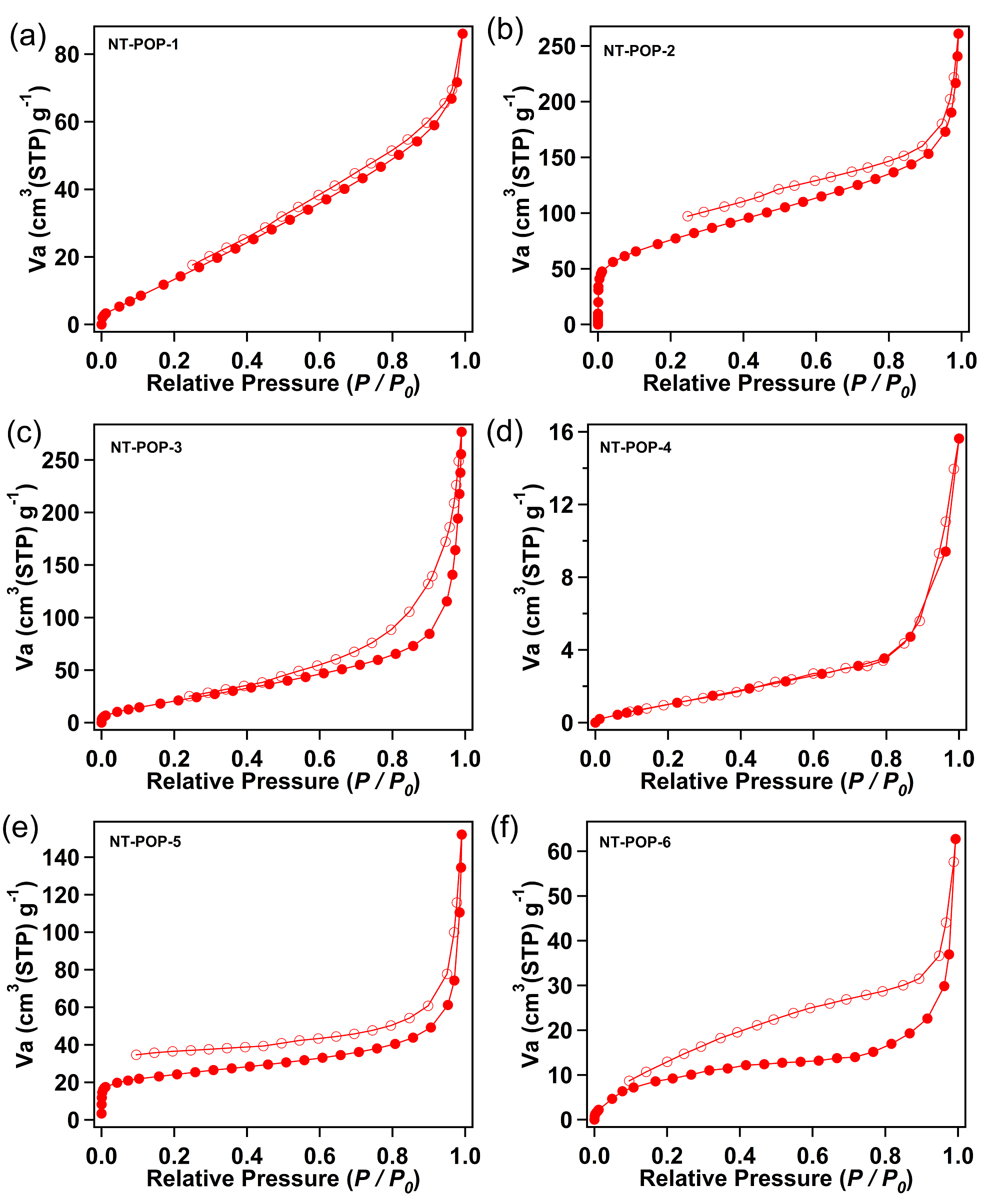
**

**Figure S1︱**Nitrogen sorption curves of NT-POPs polymers (filled circles: adsorption, open circles: desorption, STP = standard temperature pressure).

**Section D. FT-IR Spectra**

**
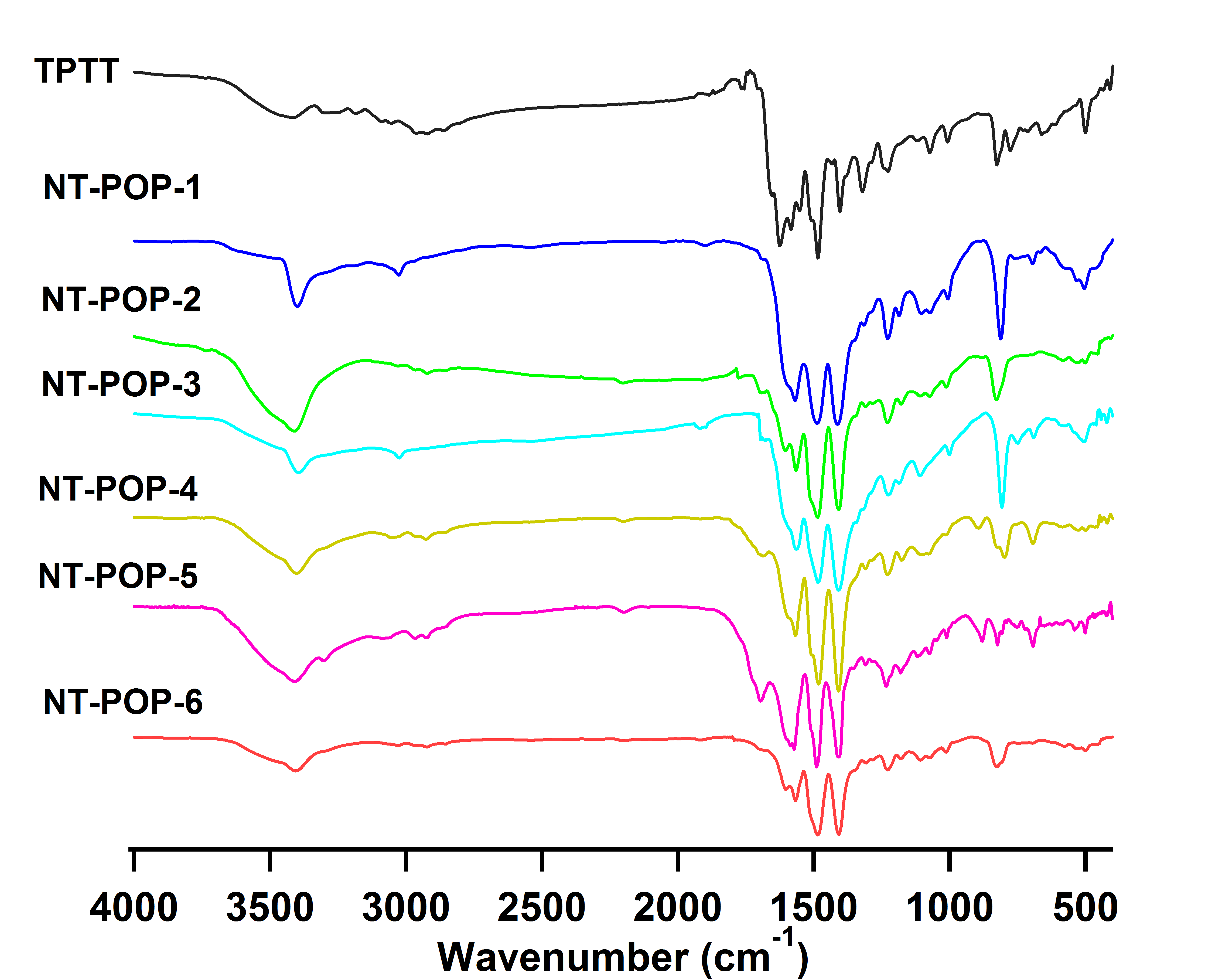
**

**Figure S2︱**FT-IR spectra of the NT-POPs porous polymers.

**Section E. TGA curves**

**
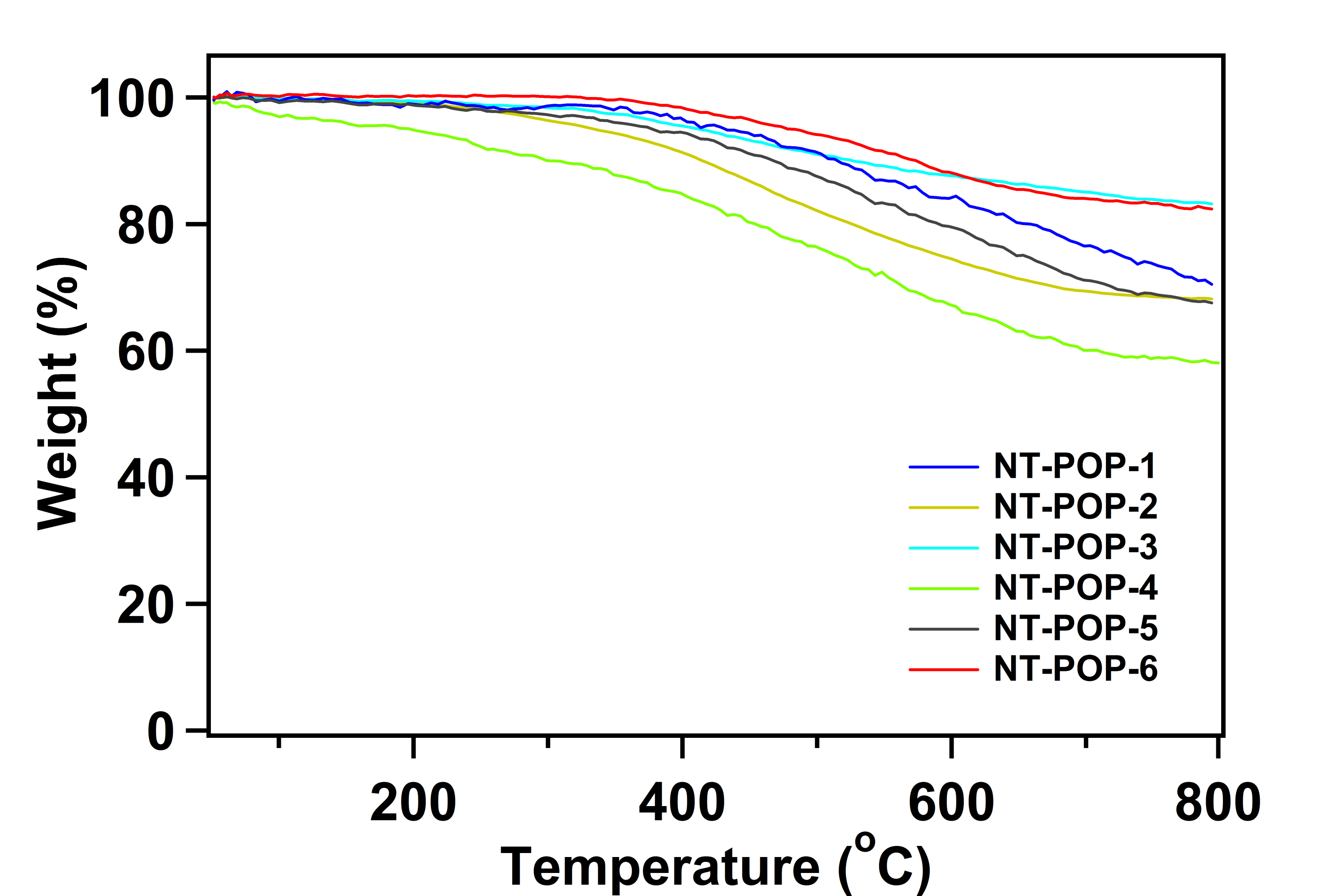
**

**Figure S3︱**TGA curves of NT-POPs.

**Section F. PXRD curves**

**
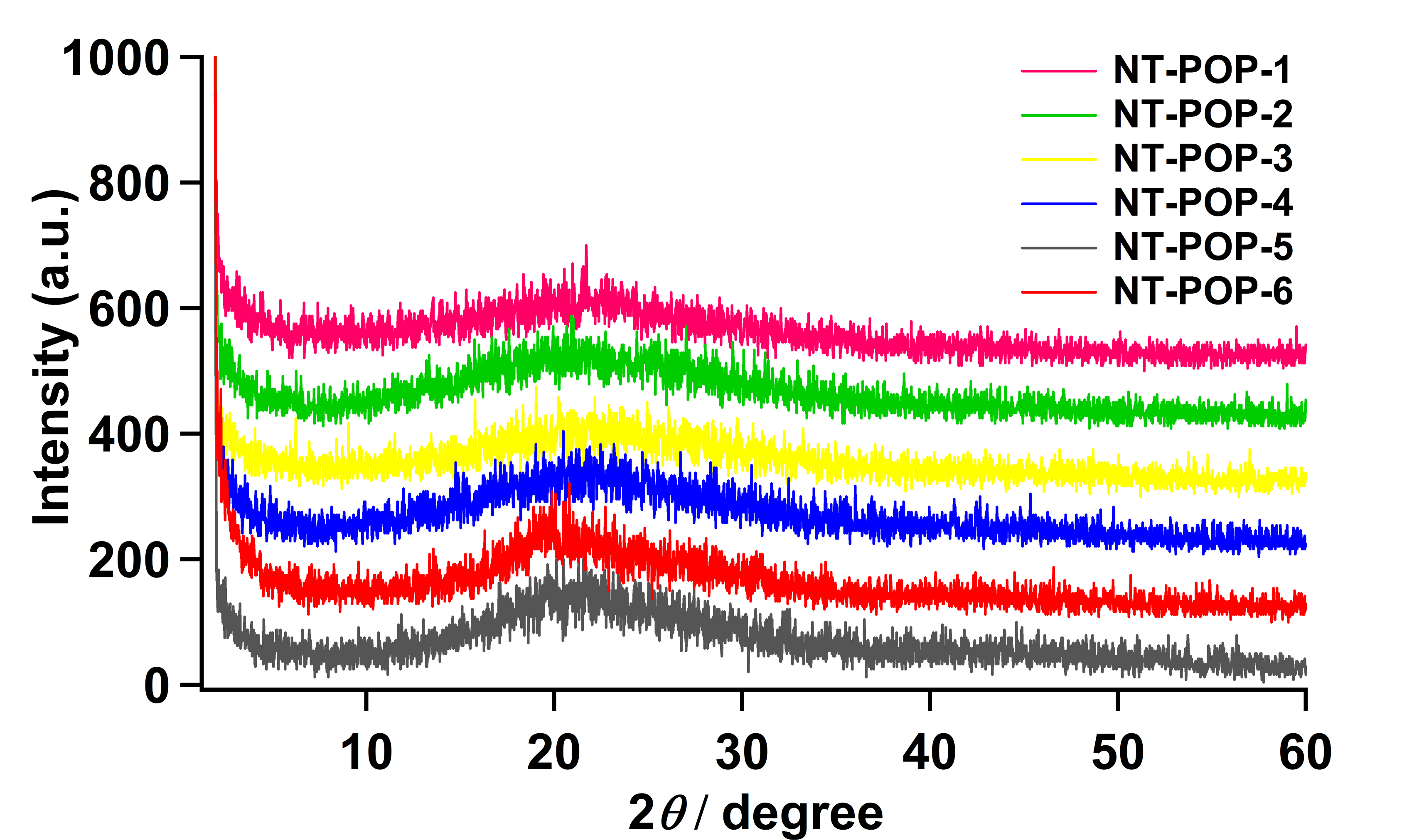
**

**Figure S4︱**Powder X-ray diffraction profiles of NT-POP-1-6.

**
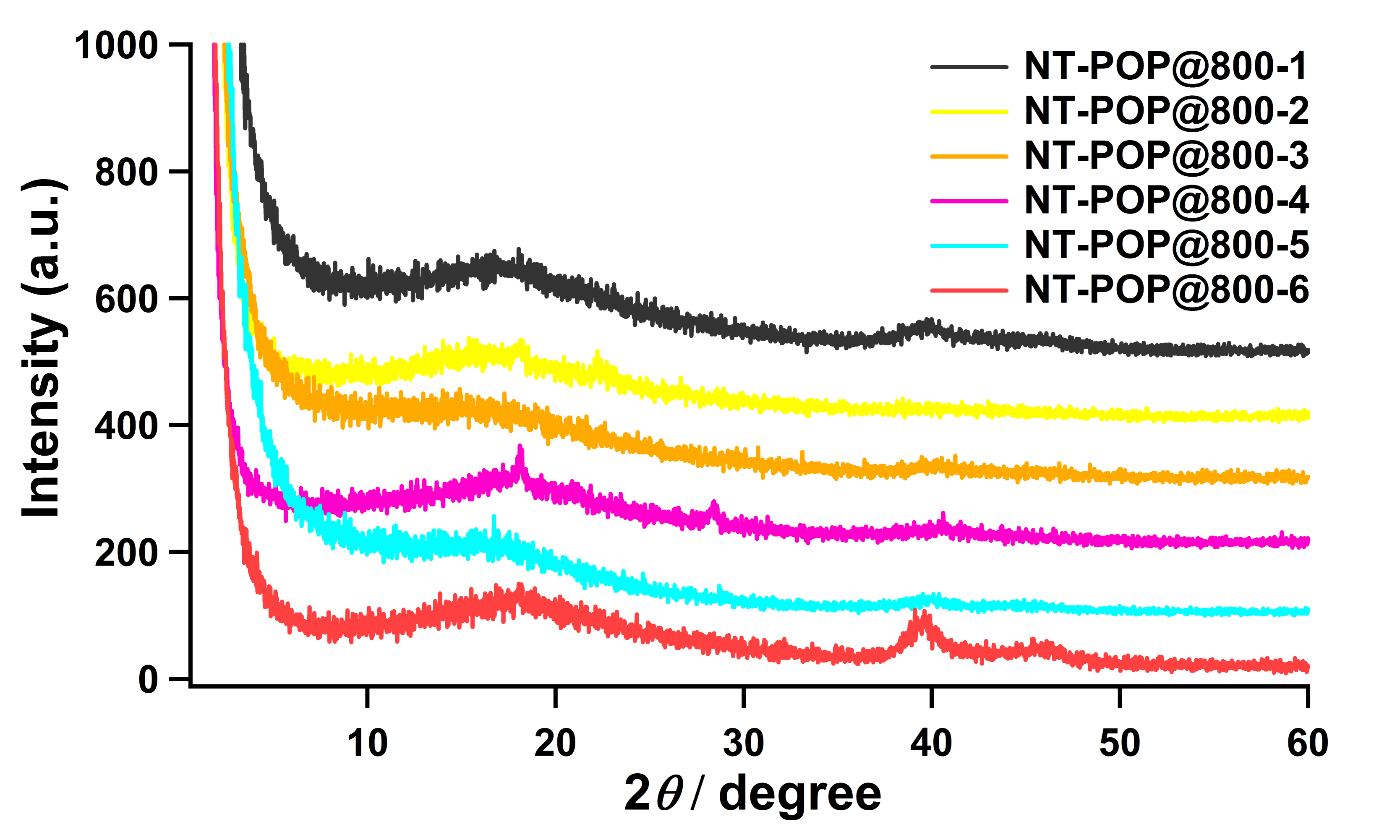
**

**Figure S5︱**Powder X-ray diffraction profiles of NT-POP@800-1-6.

**Section G. HR-TEM images**

**
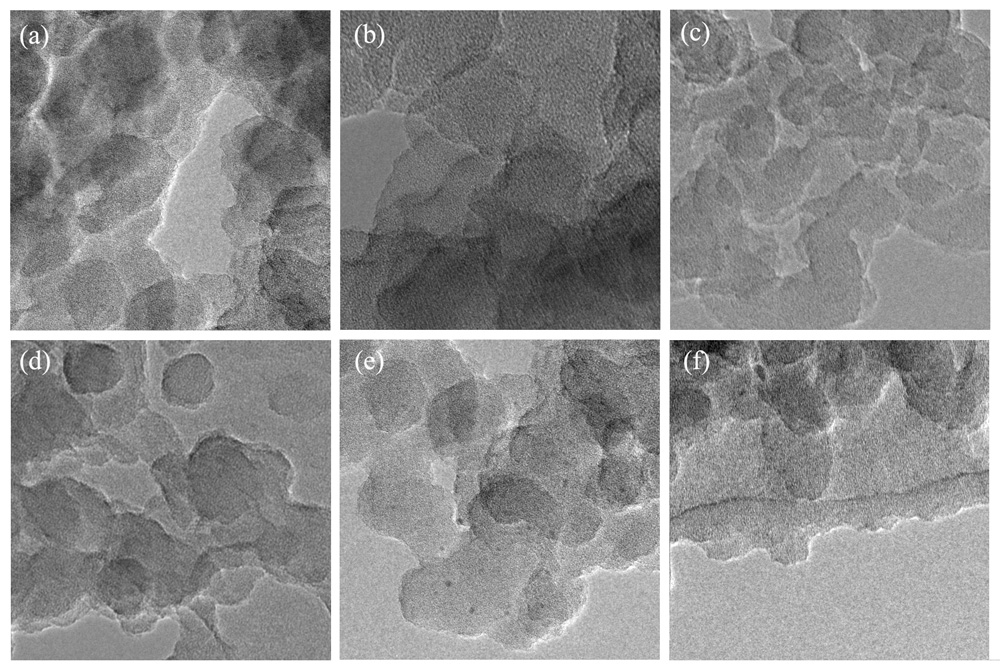
**

**Figure S6︱**HR-TEM images of (a) NT-POP@800-1, (b) NT-POP@800-2, (c) NT-POP@800-3, (d) NT-POP@800-4, (e) NT-POP@800-5, and (f) NT-POP@800-6 (scale bar 20 nm), respectively.

**Section H. FE-SEM images**


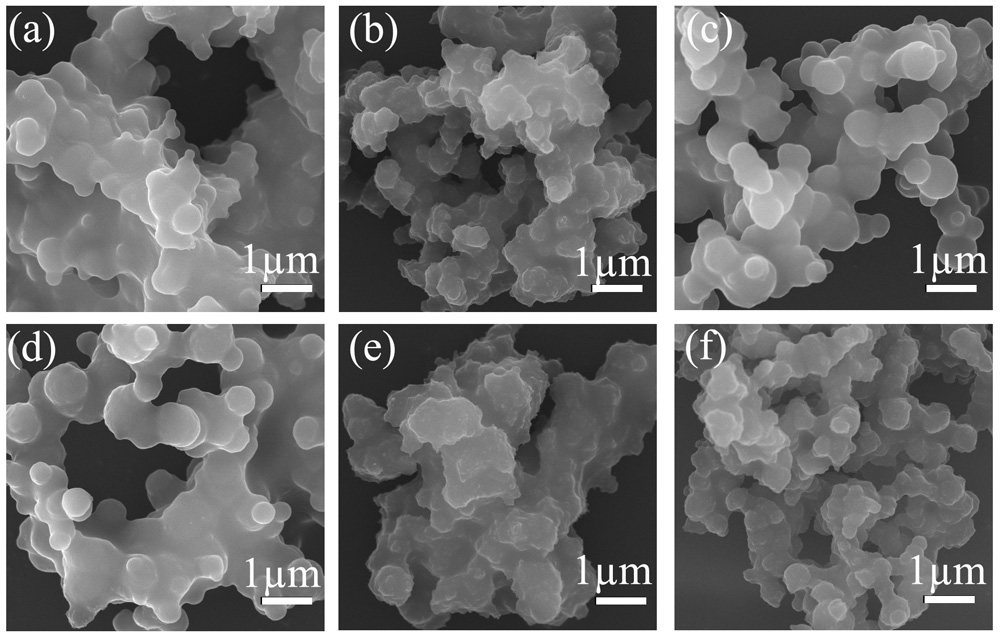


**Figure S7︱**FE-SEM images of (a) NT-POP-1, (b) NT-POP-2, (c) NT-POP-3, (d) NT-POP-4, (e) NT-POP-5, and (f) NT-POP-6, respectively.

**Section I. XPS Spectra**


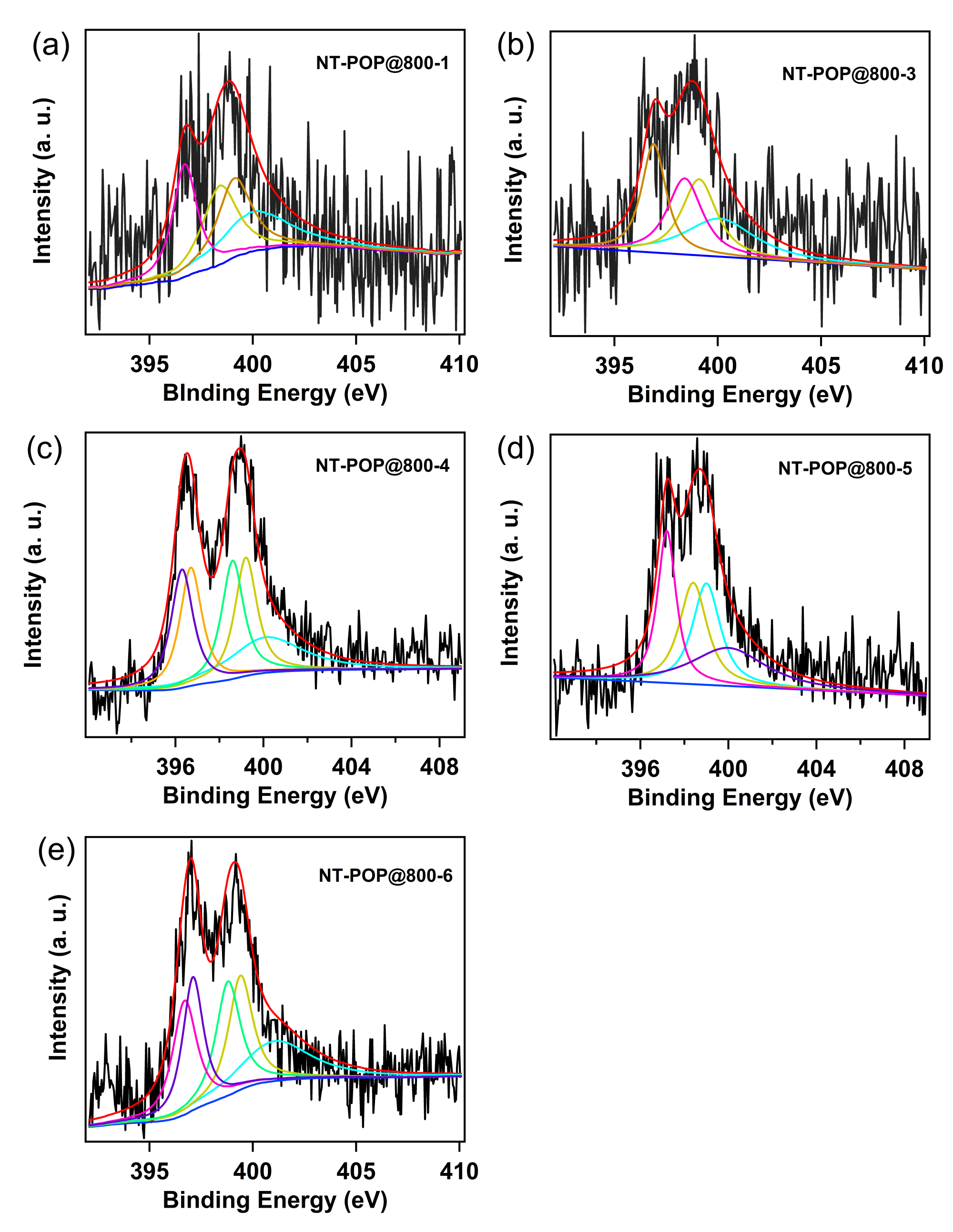


**Figure S8︱**Deconvoluted N1s spectra of (a) NT-POP@800-1, (b) NT-POP@800-3, (c) NT-POP@800-4, (d) NT-POP@800-5, and (e) NT-POP@800-6, respectively.

**Section J. Gas Adsorption Isotherms**

**
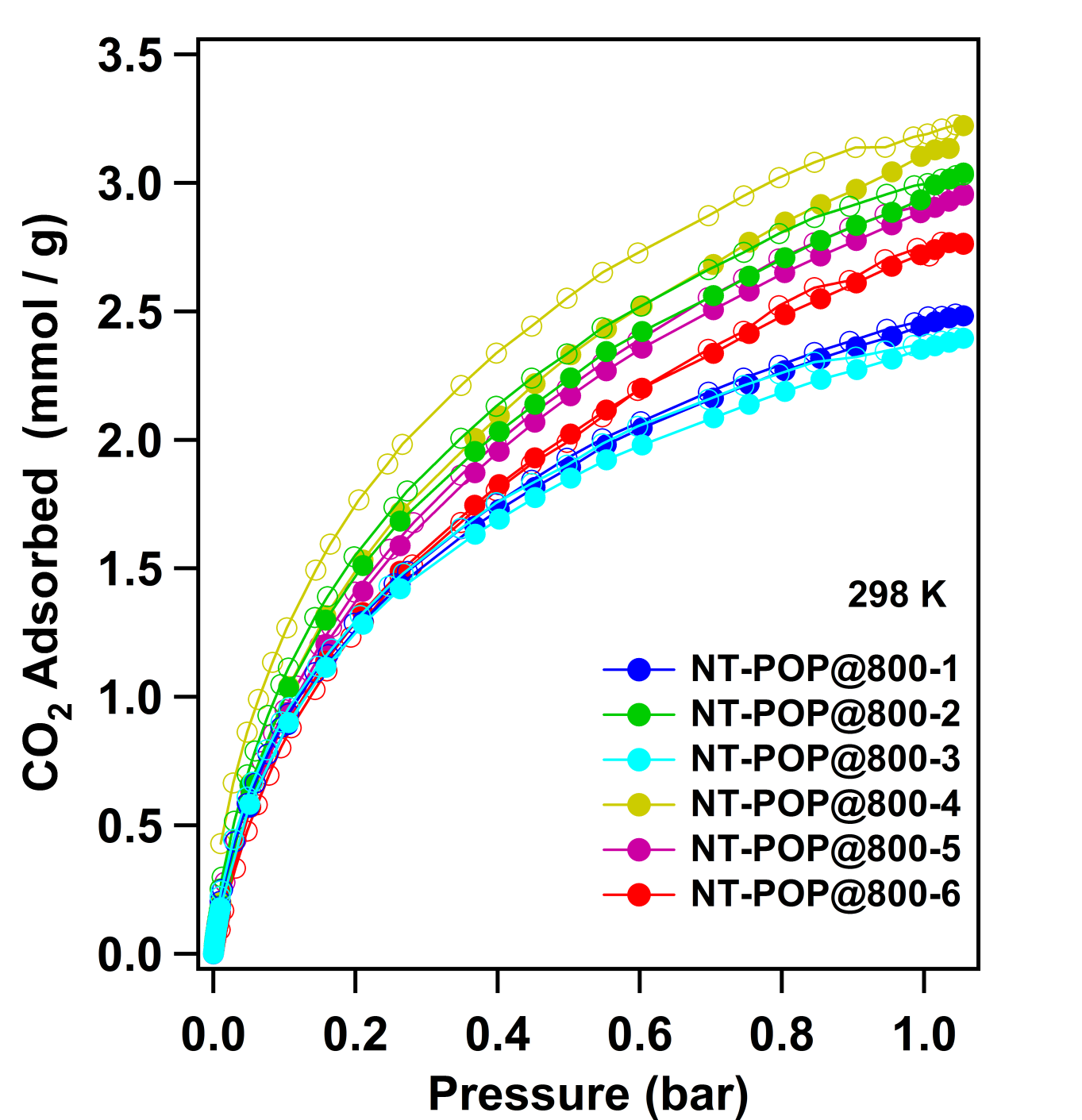
**

**Figure S9︱**CO_2_ adsorption isotherms collected at 298 K and 1.05 bar.

**
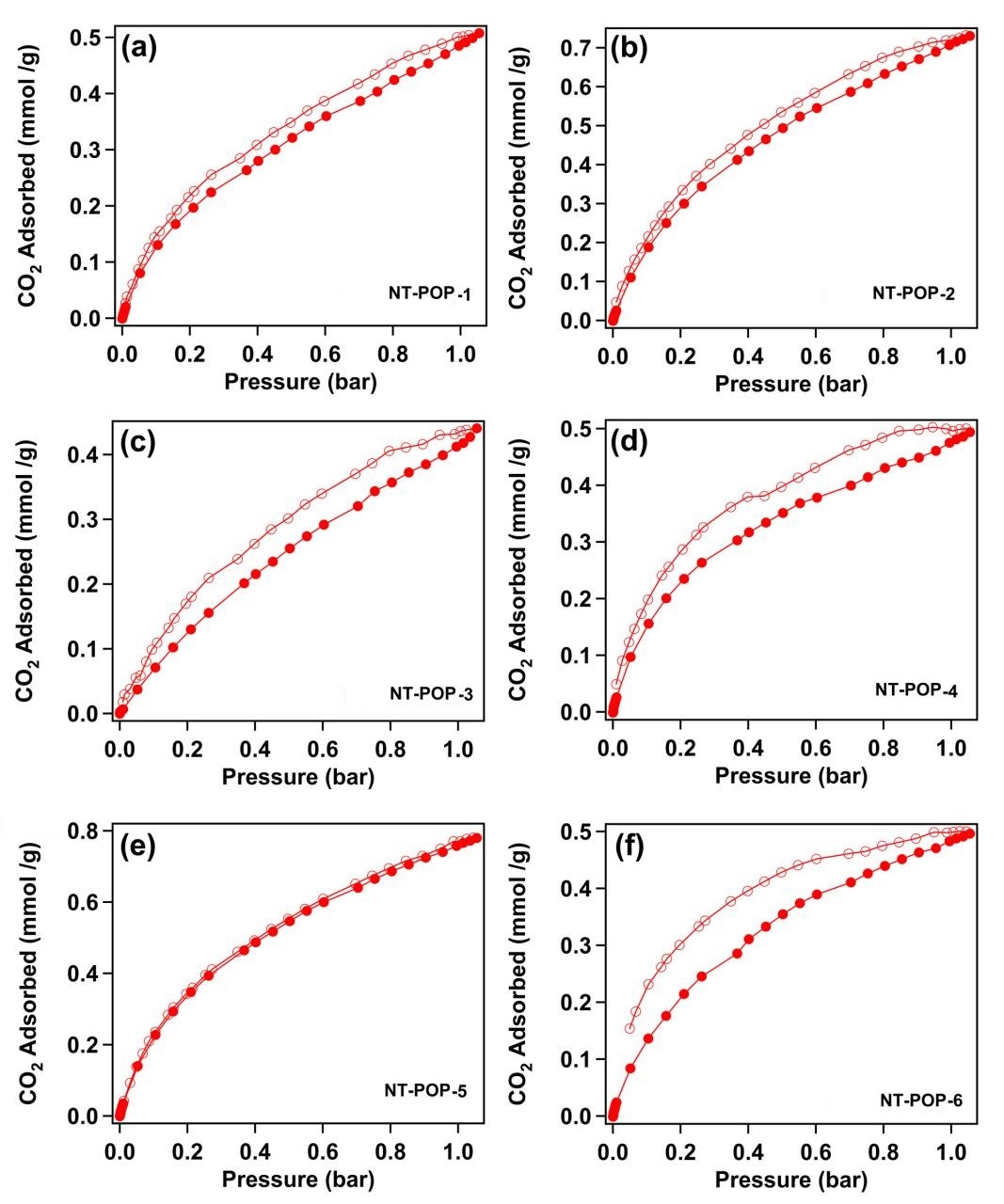
**

**Figure S10︱**CO_2_ adsorption isotherms of polymers NT-POP-1-6 at 273 K and 1.05 bar.

**
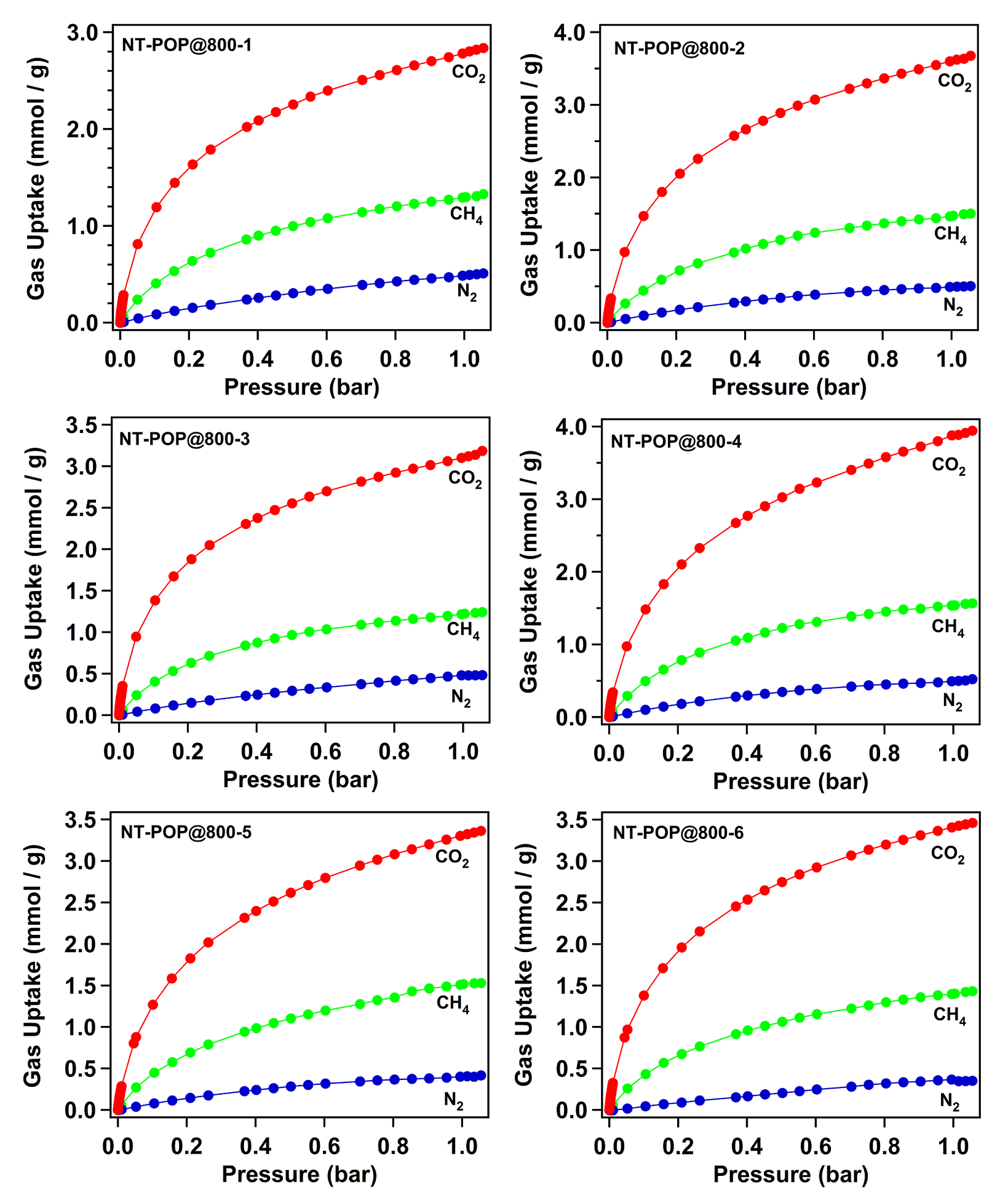
**

**Figure S11︱**Gas adsorption isotherms of polymers NT-POP@800 at 273 K and 1.05 bar.

**Section K. Gas Selectivity Analyses**

**
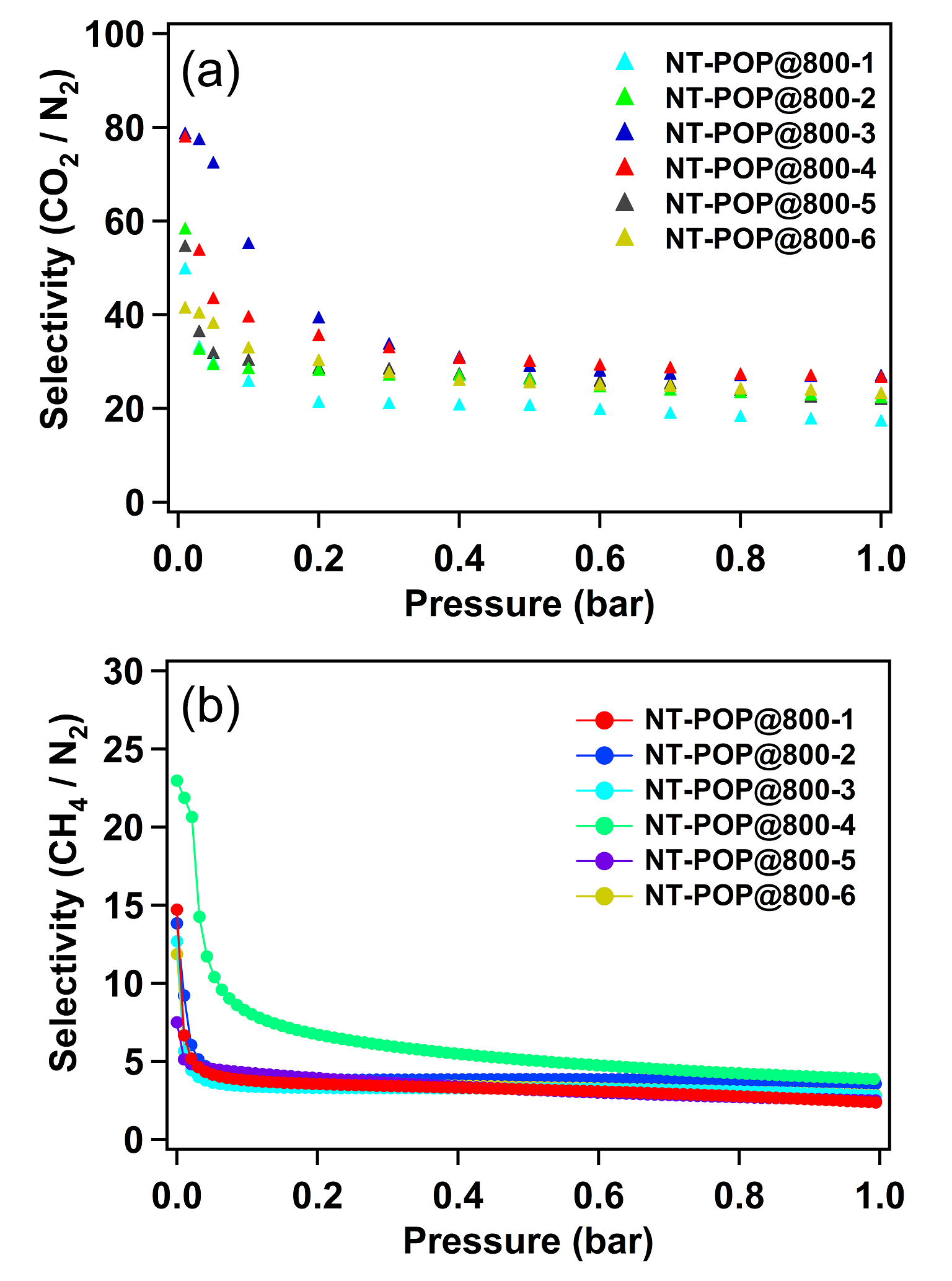
**

**Figure S12︱**(a) CO_2_/N_2_ selectivity of NT-POP@800-1-6 for a molar ratio of 15/85 at 273 K; (b) CH_4_/N_2_ selectivity of NT-POP@800-1-6 for a molar ratio of 50/50 at 273 K.

**Section L. Iodine Capture Analyses**

**(1) Recyclability for Iodine Uptake**


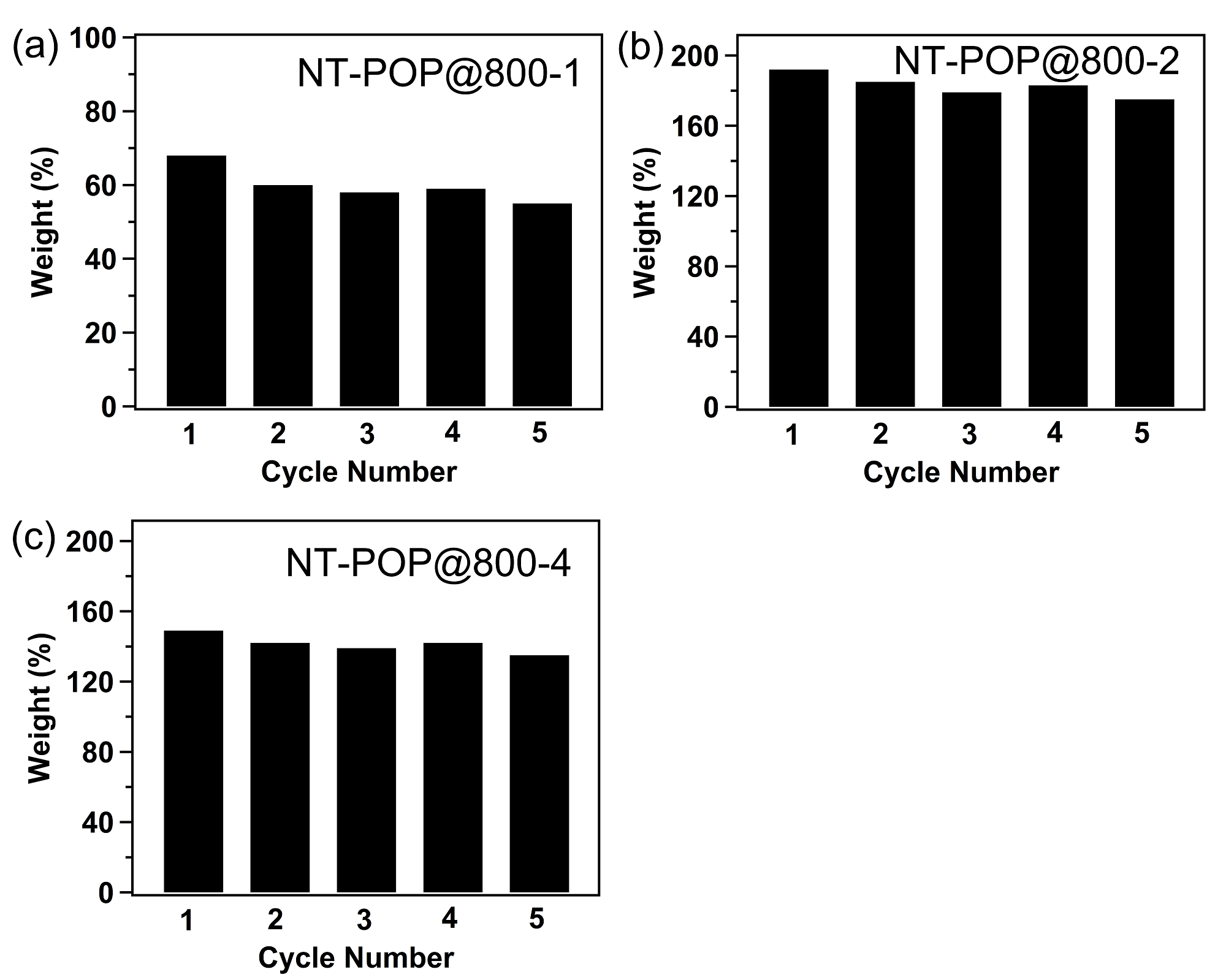


**Figure S13︱**Reusability of NT-POP@800 for iodine adsorption by vapor sublimation.

**(2) XPS Spectra**


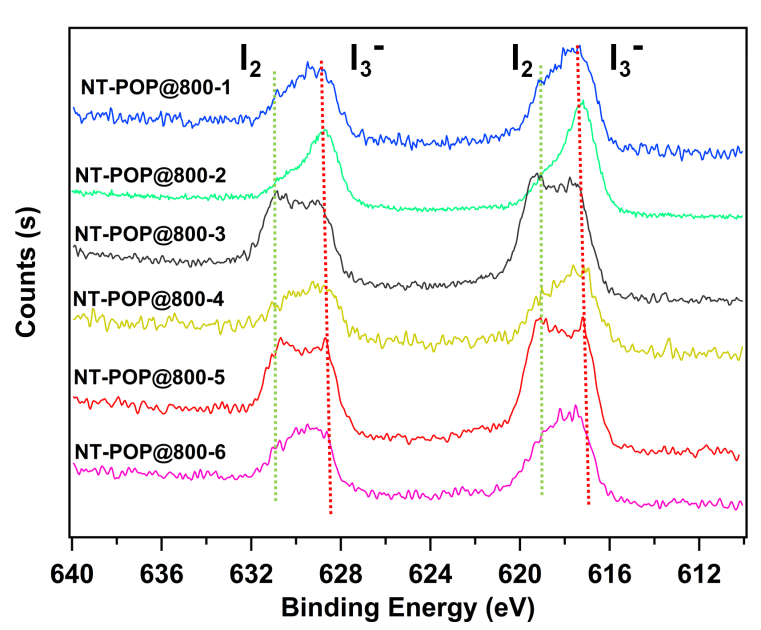


**Figure S14︱**XPS spectra of NT-POP@800-1-6 after iodine capture.

**
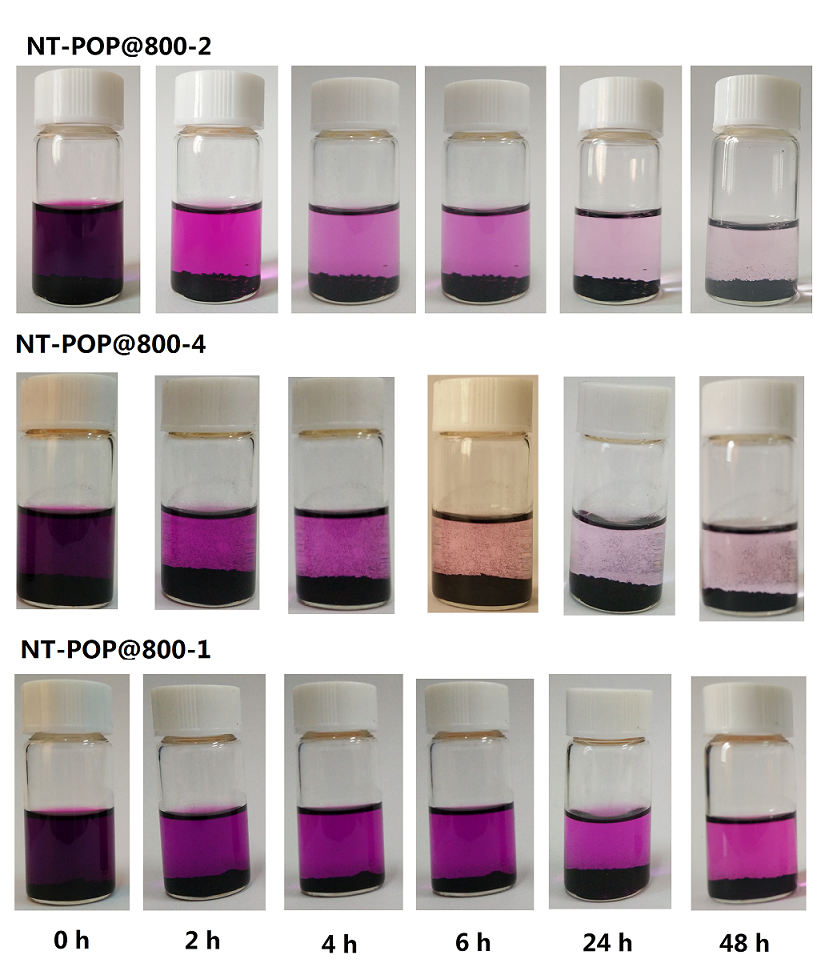
**

**Figure S15︱**The photographs show the different iodine adsorption rates of NT-POP@800.

**
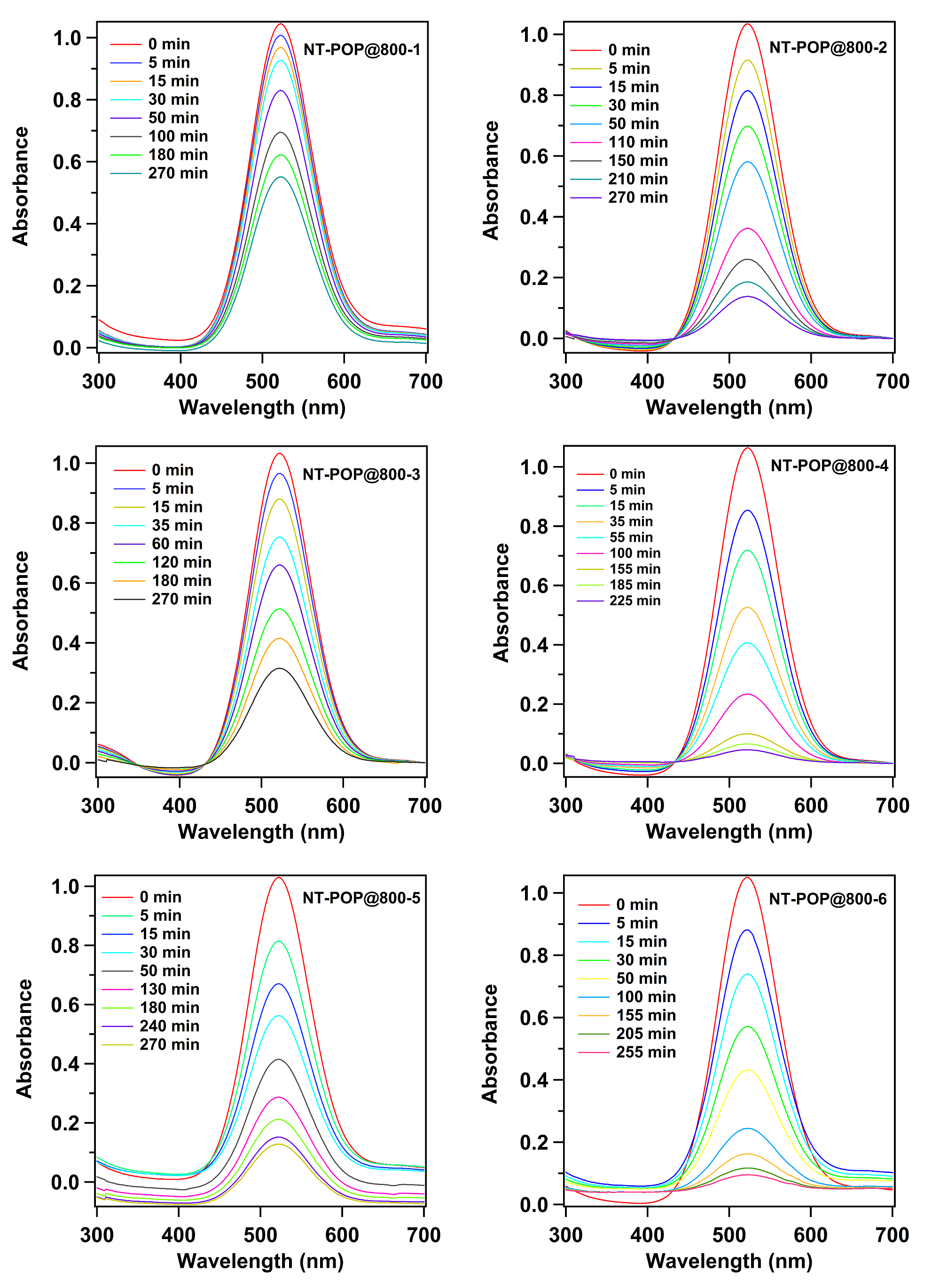
**

**Figure S16︱**UV/Vis spectra upon immersion of 30 mg NT-POP@800 in hexane solution of I_2_ (10^–2^ M). All experiments were performed at ambient temperature and pressure.

**(3) Sorption Kinetic Studies**

**The linear form of the pseudo-first-order kinetic model:**


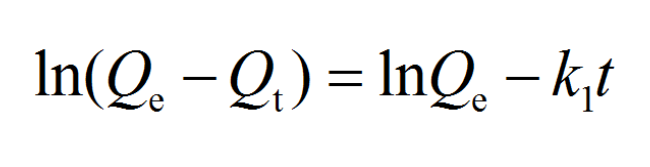


Where *Q*_t_ and *Q*_e_ are the mass percent of iodine adsorbed at time t and equilibrium (%), *k*_1_ is the pseudo-first-order rate constant of adsorption process (h^-1^).

**The linear form of the pseudo-second-order kinetic model:**


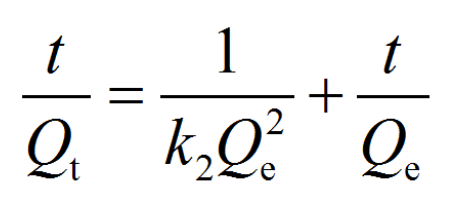


Where *Q*_t_ and *Q*_e_ are the mass percent of iodine adsorbed at time t and equilibrium (%), *k*_2_ is the pseudo-second-order rate constant of adsorption process ((% h)^-1^).


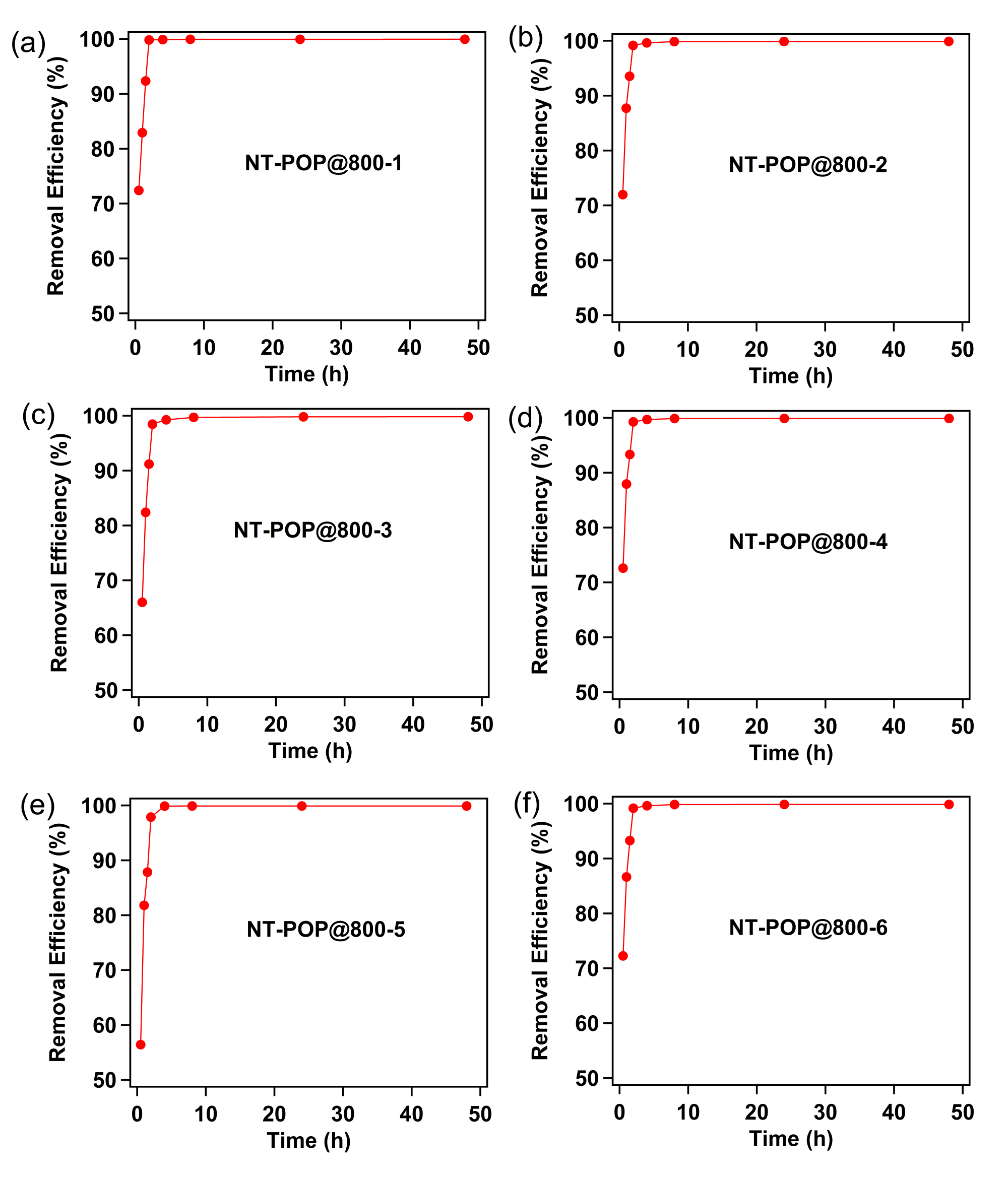


**Figure S17︱**Kinetic studies of iodine adsorption by immersing NT-POP@800-1-6 in hexane solution (4 mg mL^-1^).

**
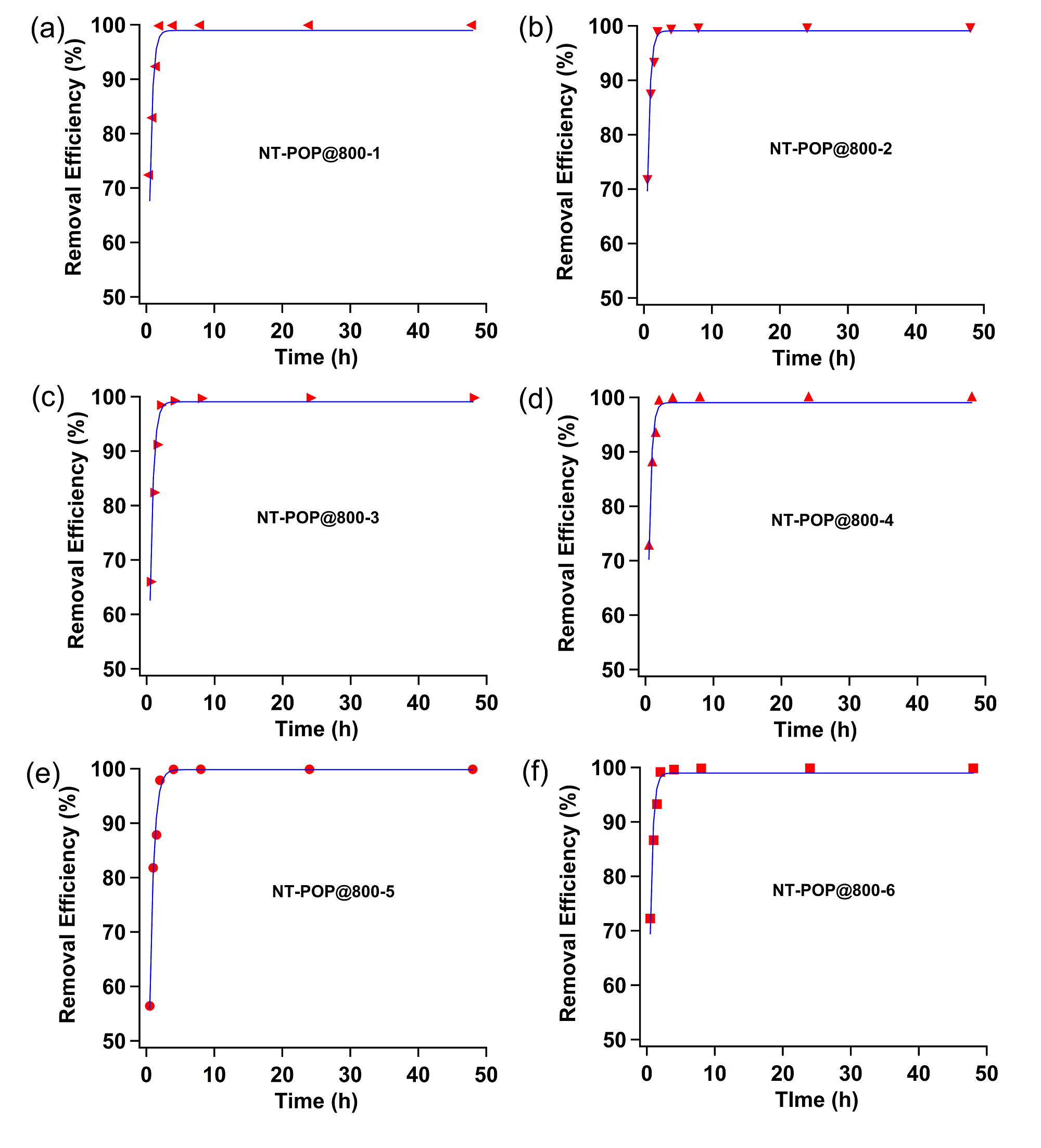
**

**Figure S18︱**The iodine adsorption kinetic was analyzed by the pseudo-first-order model. Initial concentration of iodine solution: 4 mg mL^-1^.


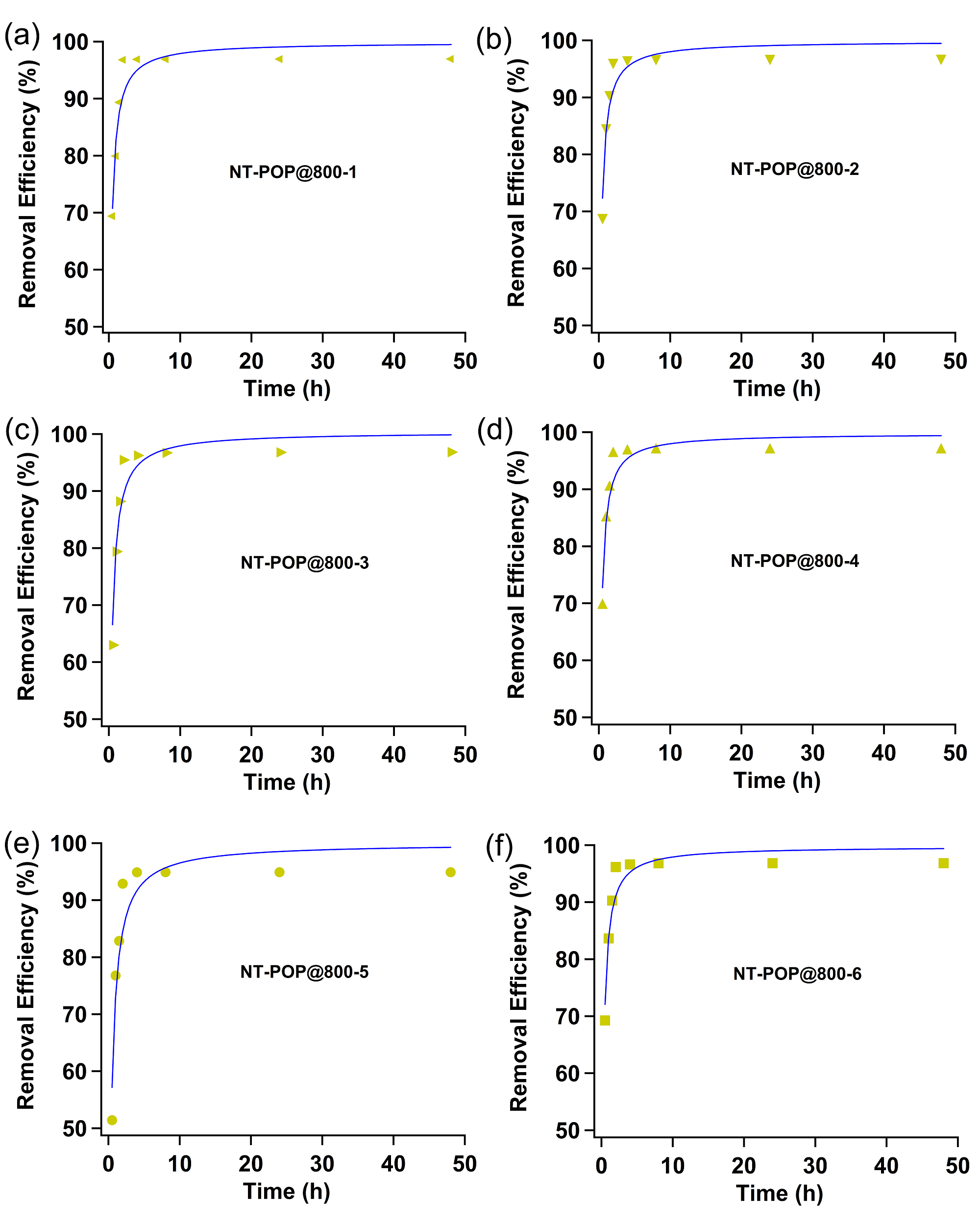

**Figure S19︱**The iodine adsorption kinetic was analyzed by the pseudo-second-order model. Initial concentration of iodine solution: 4 mg mL^-1^.

**Table S2︱Parameters of the different isotherm models extracted from the pseudo-first-order model and the pseudo-second-order model for NT-POP@800.**

| **Adsorbent** | **Pseudo-first-order** | | | **Pseudo-second-order** | | |
| --- | --- | --- | --- | --- | --- | --- |
|  | *k*_1_ (1/h) | *Q*_e_ (%) | *R*^2^ | *k*_2_ (1/h) | *Q*_e_ (%) | *R*^2^ |
| **NT-POP@800-1** | 2.3046 | 98.9578 | 0.9786 | 0.0492 | 102.9017 | 0.9002 |
| **NT-POP@800-2** | 2.4307 | 99.0872 | 0.9594 | 0.0532 | 102.8661 | 0.91 |
| **NT-POP@800-3** | 2.0004 | 99.0709 | 0.9623 | 0.0398 | 103.3831 | 0.914 |
| **NT-POP@800-4** | 2.4722 | 99.0408 | 0.9517 | 0.0545 | 102.786 | 0.9123 |
| **NT-POP@800-5** | 1.6041 | 99.9487 | 0.9878 | 0.0276 | 105.0178 | 0.9019 |
| **NT-POP@800-6** | 2.4186 | 98.9755 | 0.9533 | 0.0528 | 102.784 | 0.9151 |

**(4) Adsorption Isotherm Models
The Langmuir isotherm model:**


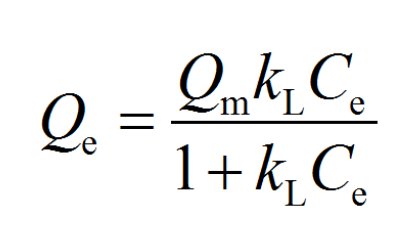


**The Freündlich isotherm model:**


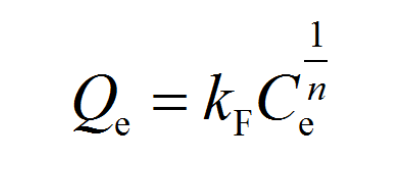


Where *k*_L_ (mg^-1^) and *Q*_m_ (mg g^-1^) are the Langmuir isotherm constants; *k*_F_ (mg^-1^) is the Freündlich isotherm constant; *C*_e_ is the concentration at equilibrium (mg mL^-1^), *Q*_e_ is the amount of iodine adsorbed at equilibrium (mg g^-1^).

**Table S3︱Parameters of two simulation models extracted from experimental adsorption isotherms data for NT-POP@800.**

| **Adsorbent** | **Langmuir isotherm** | | | **Freundlich isotherm** | | |
| --- | --- | --- | --- | --- | --- | --- |
|  | Q_m_ (mg/g) | *k*_L_(l/mg) | *R*^2^ | *k*_F_ (l/mg) | n | *R*^2^ |
| **NT-POP@800-2** | 2467 | 0.0519 | 0.9889 | 167.672 | 1.456 | 0.9712 |
| **NT-POP@800-3** | 1534 | 0.0955 | 0.9765 | 198.013 | 1.784 | 0.9347 |
| **NT-POP@800-4** | 2424 | 0.0546 | 0.9853 | 173.431 | 1.474 | 0.9639 |

**
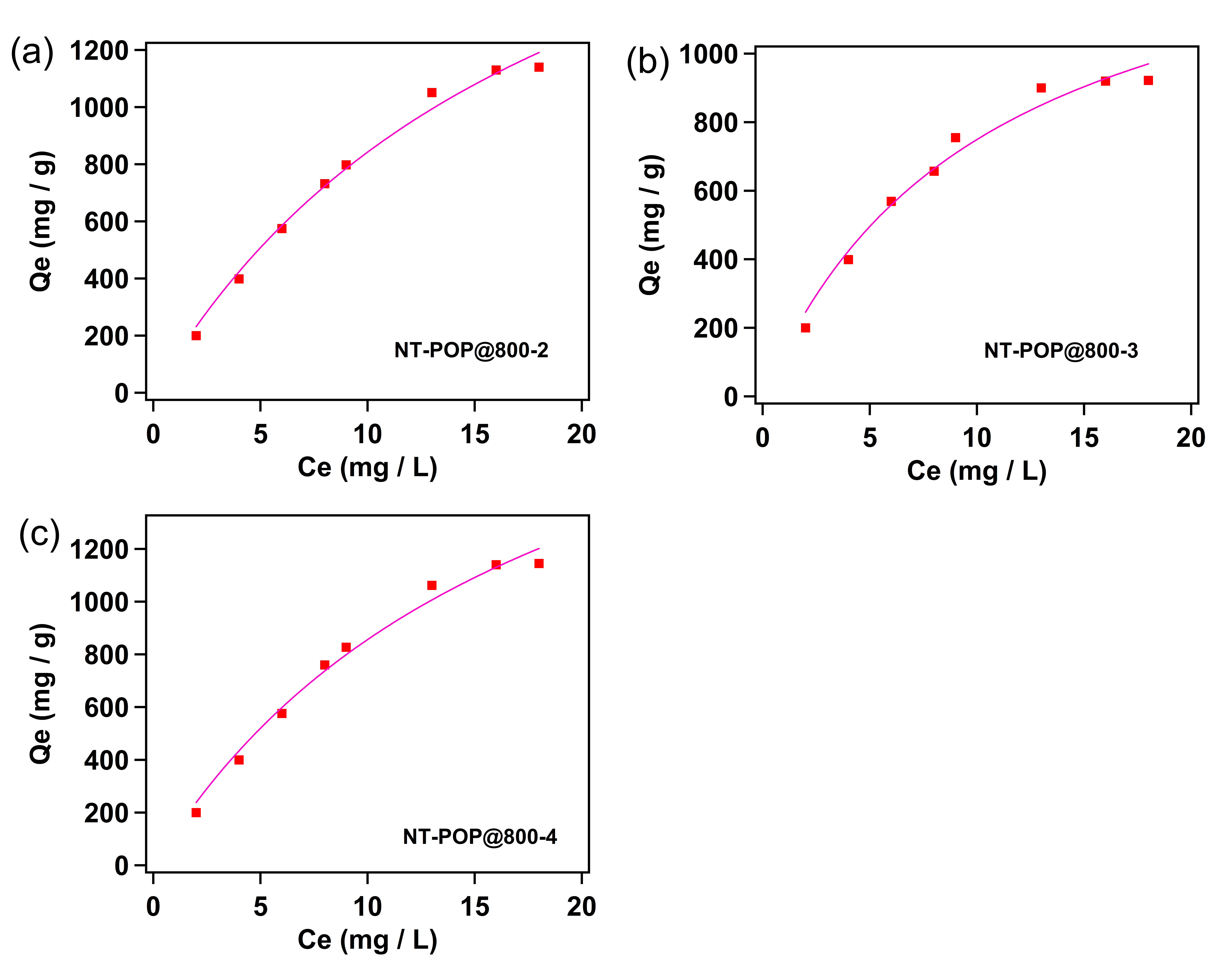
**

**Figure S20︱**Adsorption isotherms of NT-POP@800 for iodine (30 mg polymer was soaked in 3 mL of iodine solution at various concentrations for 48 h). Fitting curve: Langmuir.


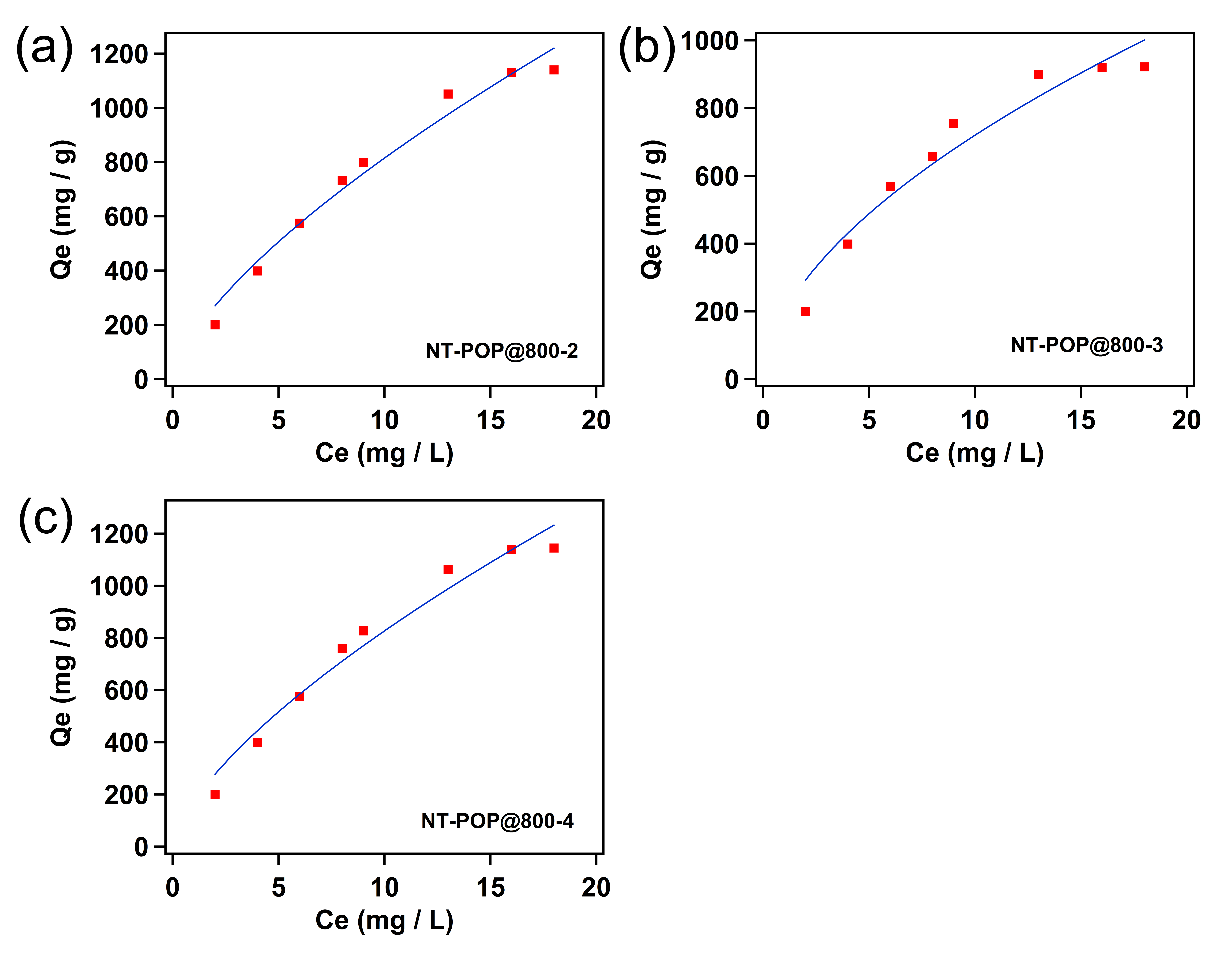


**Figure S21︱**Adsorption isotherms of NT-POP@800 for iodine (30 mg polymer was soaked in 3 mL of iodine solution at various concentrations for 48 h). Fitting curve: Freundlich.

**Section M. Supporting References**

S1. Schultz, A., Laschat, S., Diele, S. & Nimtz, M. Tetraphenylethene-derived columnar liquid crystals and their oxidative photocyclization. *Eur. J. Org. Chem.* 2829-2839 (2003).

S2. Wang, J., Mei, J., Zhao, E., Song, Z., Qin, A., Sun, J. Z. & Tang, B. Z. Ethynyl-capped hyperbranched conjugated polytriazole: click polymerization, clickable modification, and aggregation-enhanced emission. *Macromolecules* **45**, 7692-7703 (2012).
